# Ribosome State Distributions Define *Escherichia coli* Persister Physiology: Links to Formation, Stress Responses, and Resuscitation Dynamics

**DOI:** 10.1111/1751-7915.70352

**Published:** 2026-04-13

**Authors:** Hyein Kim, Sooyeon Song

**Affiliations:** ^1^ Department of Animal Science Jeonbuk National University Jeonju‐Si Jellabuk‐Do South Korea; ^2^ Agriculture Convergence Technology Jeonbuk National University Jeonju‐Si Jellabuk‐Do South Korea

**Keywords:** 100S ribosome, antibiotic persistence, bacterial resuscitation, ribosome hibernation, ribosome sedimentation profiling

## Abstract

Persister cells survive antibiotic exposure and contribute to infection relapse, yet the molecular features that distinguish them from actively growing cells remain incompletely defined. Here, we used sucrose gradient‐based ribosome sedimentation profiling to characterise ribosome complex distributions in *Escherichia coli* persister cells and monitored their dynamics during resuscitation. Rifampicin‐induced persister cells were characterised by pronounced enrichment of translationally inactive 90–100S ribosome complexes and a concomitant reduction in 70S ribosomes relative to exponentially growing cells. Upon nutrient replenishment, ribosome distributions progressively shifted toward higher 70S and polysome (complexes of multiple ribosomes simultaneously translating a single mRNA) levels, coinciding with growth recovery, indicating that resuscitation involves gradual remodelling of ribosome states rather than abrupt restoration of active translation. Functional analysis of ribosome‐associated factors demonstrated that RMF, HPF and RaiA promote 100S ribosome accumulation and enhance persister formation, whereas deletion of *rmf* severely impaired both 100S formation and persistence. In contrast, loss of HflX did not measurably affect persister formation, consistent with a role downstream of persister establishment. In multiple stress‐induced persister models including rifampicin, tetracycline, CCCP and starvation, as well as in a clinically relevant *E. coli* O157:H7 (EHEC) strain, ribosome distributions consistently exhibited a quantitative reversal of the AUC_70S/AUC_100S ratio (Ratio < 1.0) relative to exponentially growing cells (Ratio > 1.0). Collectively, these findings demonstrate that this shift in the 70S‐to‐100S balance is a consistent and shared feature of *E. coli* persister physiology and that ribosome state distributions link persister formation to resuscitation dynamics. These findings provide a quantitative ribosome‐state framework that may inform the development of anti‐persistence strategies targeting ribosome hibernation factors.

## Graphical abstract

The AUCS_70_/AUCS_100_ ratio, derived from ribosome sedimentation profiling, quantitatively distinguishes *Escherichia coli* persisters from growing cells across multiple stress conditions and identifies RMF as the primary target for anti‐persistence strategies.

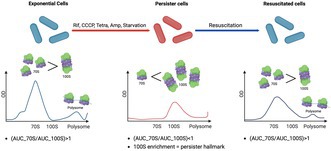

## 1 Introduction

The global rise in antibiotic consumption has accelerated the emergence and dissemination of antibiotic‐resistant pathogens and is associated with increasing mortality worldwide (Van Boeckel et al. 2014; O'Neill 2016; Cassini et al. 2019). Bacterial populations can survive antibiotic exposure through three distinct mechanisms: resistance, tolerance and persistence. Resistance is driven by heritable genetic changes that increase the minimum inhibitory concentration (MIC), whereas tolerance and persistence do not require an elevated MIC but instead alter killing dynamics. Critically, tolerance manifests as a population‐wide reduction in killing rate, whereas persistence is characterised by the survival of a rare phenotypic subpopulation exhibiting biphasic killing kinetics—a distinction with important implications for treatment outcomes (Johnson and Levin 2013; Brauner et al. 2016).

Persister cells are phenotypic variants that transiently reprogram their physiology in response to stress and are widely recognised as a major contributor to chronic and relapsing infections (Hobby et al. 1942; Bigger 1944; Kunnath et al. 2024). Persister cells typically comprise a minor fraction of the population (commonly < 1%, depending on growth conditions) and, upon stress removal, resume growth to regenerate a population genetically indistinguishable from the parental cells (Hofsteenge et al. 2013; Stepanyan et al. 2015; Nakouti et al. 2017).

Multiple, partially overlapping pathways have been implicated in persister formation, including toxin–antitoxin modules (Fernández‐García, Kirigo, et al. 2024), the SOS response, global stress regulators and the alarmone (p)ppGpp (Niu et al. 2024). Stress leads to the accumulation of (p)ppGpp, which interacts with RNA polymerase to increase expression of RpoS, a master regulator of the general stress response (Jishage et al. 2002). Together, (p)ppGpp and RpoS suppress ribosomal RNA and protein synthesis and reduce translational activity, driving cells into a dormant state characteristic of persister formation (Chatterji and Ojha 2001; Cho et al. 2015). Despite their mechanistic diversity, these pathways converge on a unifying physiological theme: the suppression of translation and remodelling of ribosome states under stress (Cho et al. 2015; Song and Wood 2020; Blattman et al. 2024).

Upon restoration of growth‐permissive conditions, persister cells can restore metabolic activity and resuscitate (Yamasaki et al. 2020). Nutrient sensing through chemotaxis, for example to alanine or glucose, initiates signalling cascades that reduce intracellular cAMP levels and promote recovery of hibernated ribosomes (Yamasaki et al. 2020). While (p)ppGpp is essential for persister formation, it does not appear to play a direct role during resuscitation (Song and Wood 2020). Instead, ribosome‐associated factors such as RMF, HPF and RaiA suppress resuscitation by promoting ribosome inactivation and dimerisation, whereas the ribosome rescue factor HflX facilitates resuscitation by dissociating inactive ribosome complexes upon stress removal (Song and Wood 2020). Consistent with the view that translational capacity constrains resuscitation, higher intracellular ribosome content has been associated with faster reactivation of persister cells (Kim, Yamasaki, et al. 2018), indicating that both ribosome abundance and the distribution of ribosomes across translation‐active and ‐inactive states shape resuscitation capacity.

Recent work has further underscored a close relationship between persister dormancy and ribosome remodelling (Cho et al. 2015; Kim, Yamasaki, et al. 2018; Song and Wood 2020). During persister formation, ribosomes can undergo extensive disassembly into inactive subunits accompanied by degradation of a large fraction of rRNA and tRNA, while a subset of ribosomes persists in a structurally protected form (Cho et al. 2015). Lang et al. further demonstrated that persister cells retain non‐translating 70S ribosomes in a protected state termed “sleeping ribosomes,” consistent with the 100S ribosome dimers originally described in stationary‐phase *Escherichia coli* (Wada et al. 1990; Lang et al. 2021). The functions of RMF, HPF and RaiA in promoting ribosome inactivation and 100S dimer formation were subsequently incorporated into the ppGpp ribosome dimerisation persister (PRDP) model, which positions ribosome inactivation and reactivation as central events in persister formation and resuscitation (Song and Wood 2020).

According to the PRDP model, stress‐induced (p)ppGpp accumulation upregulates RaiA, RMF and HPF, thereby stabilising translation‐inactive ribosome complexes (Song and Wood 2020). RaiA transiently inactivates 70S ribosomes, while RMF and HPF cooperatively facilitate their conversion into hibernating 100S dimers that are translation‐incompetent (Polikanov et al. 2012; Lang et al. 2021). Maintenance of these inactive ribosome pools supports survival during dormancy. Upon stress removal, reduced cAMP levels activate ribosome rescue factors such as HflX, enabling dissociation of 100S dimers into active 70S monomers and restoration of translation (Yoshida and Wada 2014; Song and Wood 2020). Sucrose density gradient‐based ribosome sedimentation profiling, including glutaraldehyde‐stabilised conditions that preserve higher‐order ribosome complexes, provides a practical and quantitative tool for resolving and comparing these states by fractionating 30S, 50S, 70S, 100S and polysome complexes.

Despite these advances, the functional contributions of distinct ribosome complex distributions to persister formation, survival and resuscitation remain incompletely defined. In particular, it is unclear whether different stress conditions generate persister subtypes characterised by distinct ribosome sedimentation profiles and whether these profiles predict resuscitation kinetics and survival outcomes. In this study, we address these questions by pursuing three objectives: (1) to define how ribosome‐associated hibernation and rescue factors reshape gradient‐resolved ribosome complex distributions in *E. coli* persister cells; (2) to quantify and compare ribosome sedimentation profiles across persister cells generated by diverse stresses; and (3) to establish a functional framework linking ribosome complex distributions to resuscitation dynamics and survival. Importantly, whereas prior studies have largely relied on qualitative or single‐condition assessments of ribosome states in persisters, this study provides the first systematic, quantitative cross‐condition comparison using AUC‐based sedimentation metrics, including the AUC_70S/AUC_100S ratio as a novel index of persister ribosome state. Beyond mechanistic insight, identifying ribosome hibernation factors as determinants of persister formation offers potential biotechnological applications, including their use as molecular targets for anti‐persistence compounds and as markers in high‐throughput screening platforms aimed at disrupting persister physiology in clinically relevant bacteria.

## 2 Materials and Methods

### 2.1 Bacterial Strains and Growth Conditions

*Escherichia coli* BW25113 wild‐type, Δ*rmf*, Δ*raiA* and Δ*hpf* knockout strains, along with the clinical strain *E. coli* O157:H7, were used in this study (Table 1). Knockout strains were obtained from the KEIO collection. In addition, *E. coli* strains harbouring the pCA24N‐empty vector or pCA24N‐*rmf* expression plasmid were acquired from the ASKA collection (Table 1). All strains were stored at −80°C in glycerol stocks. Strains were streaked on LB agar plates and incubated overnight at 37°C. A single colony was inoculated into LB broth and cultured at 37°C with shaking (250 rpm) for 16 h. The overnight culture was diluted 1:100 into fresh LB broth and grown until the OD₆₀₀ reached 0.8, indicating the exponential growth phase.

**TABLE 1:** Strains and plasmids.

Strains	Features	Source
*E. coli* BW25113	Wild type	Parental strain of the Keio collection (Baba et al. 2006)
*E. coli* BW25113 Δ*rmf*	Δ*rmf*, Km^R^	Keio collection (Baba et al. 2006)
*E. coli* BW25113 Δ*hpf*	Δ*hpf*, Km^R^	Keio collection (Baba et al. 2006)
*E. coli* BW25113 Δ*raiA*	Δ*raiA*, Km^R^	Keio collection (Baba et al. 2006)
*E. coli* BW25113 Δ*hflX*	Δ*hflX*, Km^R^	Keio collection (Baba et al. 2006)
*E. coli* O157:H7 ATCC 43889	Enterohemorrhagic *E. coli* (EHEC), Shiga toxin‐producing (stx1^+^, stx2^+^), eae^+^	American type culture collection
Plasmids		
pCA24N	Cm^R^; *lacI* ^*q*^	ASKA library (Kitagawa et al. 2005)
pCA24N_*rmf*	Cm^R^; *lacI* ^*q*^, P_T5‐lac_::*rmf* ^+^	ASKA library (Kitagawa et al. 2005)
pCA24N_*hpf*	Cm^R^; *lacI* ^*q*^, P_T5‐lac_::*hpf* ^+^	ASKA library (Kitagawa et al. 2005)
pCA24N_*raiA*	Cm^R^; *lacI* ^*q*^, P_T5‐lac_::*raiA* ^+^	ASKA library (Kitagawa et al. 2005)
pCA24N_*hflX*	Cm^R^; *lacI* ^*q*^, P_T5‐lac_::*hflX* ^+^	ASKA library (Kitagawa et al. 2005)

### 2.2 Persister Cell Formation and Resuscitation

Persister cells were generated using several induction methods. Natural persister cells were obtained by directly exposing exponential phase cultures to ampicillin (100 μg/mL) for 3 h at 37°C (Kim, Chowdhury, et al. 2018; Blattman et al. 2024). For antibiotic/chemical pre‐treatment–induced persister cells, exponential phase cells were pre‐treated with rifampicin (100 μg/mL, for 30 min), tetracycline (50 μg/mL, for 30 min) or CCCP (50 μg/mL, for 3 h) and subsequently exposed to ampicillin (100 μg/mL) for 3 h at 37°C (Kwan et al. 2013; Kim, Chowdhury, et al. 2018; Narayanaswamy et al. 2018; Blattman et al. 2024; Fernández‐García, Song, et al. 2024). The persister cells generated by rifampicin pretreatment in this study were produced using the protocol established and validated by Kwan et al. (2013), which showed that the resulting cells exhibit the defining characteristics of persister cells, including multidrug tolerance without genetic change and full restoration of antibiotic susceptibility upon regrowth.

Starvation‐induced persister cells were obtained by washing exponential cultures, resuspending them in 0.85% NaCl and incubating at 37°C for 6 days under nutrient starvation, followed by exposure to ampicillin (100 μg/mL) for 3 h (Kim, Yamasaki, et al. 2018; Blattman et al. 2024).

For resuscitation assays, persister cells from each condition were harvested and washed twice with 0.85% NaCl to remove residual antibiotics. The pellets were resuspended in fresh LB broth and incubated at 37°C. Samples were collected at 1, 2 and 3 h (BW25113) or at 1 and 3 h ( *E. coli* O157:H7) to assess time‐dependent resuscitation.

### 2.3 Preparation of Association Buffer and Sucrose Gradient

Association buffer (Buffer A) was prepared by dissolving Tris base in DEPC‐treated water. The pH was adjusted to 7.6 with HCl, followed by the addition of magnesium acetate and ammonium acetate. 2‐Mercaptoethanol was added immediately before use. The buffer was filtered through a 0.2 μm filter and stored at 4°C in light‐protected bottles. Sucrose solutions (5%, 10%, 15%, 18%, 20% and 30%) were prepared in Buffer A and stored at 4°C. For ribosome sedimentation profiling of 30S, 50S and 70S ribosomes, 0.25% glutaraldehyde was not added; however, for profiling of 70S and 100S ribosomes, 0.25% glutaraldehyde was added to the 20% sucrose solution (Yoshida et al. 2002; Kato et al. 2010).

Gradients were assembled by sequential layering of each sucrose solution into an ultracentrifuge tube, followed by snap‐freezing using a dry ice/ethanol bath. The gradients were stored at −20°C for 6 h, then thawed slowly at 4°C for 20 h prior to use (Ueta et al. 2017; Yoshida et al. 2021).

### 2.4 Preparation of Crude Ribosomes

Cell cultures (exponential or persister phase) were quenched by pouring into centrifuge tubes containing RNAlater and immediately immersed in a dry ice/ethanol bath for 30s without freezing. After centrifugation, pellets were resuspended in Buffer A containing RNAlater. Sonication was performed on ice for 2 min followed by 2 min cooling, repeated three times. The lysate was layered onto a 30% sucrose cushion in Buffer A and centrifuged at 206,000 × *g* for 3 h at 4°C using an SW41 rotor. Ribosome pellets were gently resuspended in Buffer A and stored at 4°C. RNA integrity and purity were verified by A260/A280 ratio measurements prior to further analysis. Additionally, the structural integrity of the recovered ribosome complexes was confirmed by visual inspection of the sedimentation profiles; samples showing minimal absorbance in the soluble (SOL) fractions at the top of the gradient and well‐resolved ribosomal peaks—indicative of negligible rRNA degradation (Maguire et al. 2008)—were selected for downstream quantification. Equal RNA concentrations were applied consistently across all experimental conditions, following the normalisation approach established in previous ribosome sedimentation profiling studies (Kato et al. 2010; Ueta et al. 2017). Ribosome concentrations were determined using a microplate reader by measuring absorbance at 260 nm. Based on the measured RNA concentration, ribosome samples were diluted with ribosome buffer to a final concentration of 3000 ng/μL. This normalisation was applied consistently across all samples to ensure equal ribosome input among experimental conditions.

### 2.5 Ribosome Analysis by Sucrose Density Gradient Centrifugation

Normalised ribosome samples were layered on top of preformed 5%–20% sucrose gradients in Buffer A and centrifuged at 285,000 × *g* (40,000 rpm) for 1.5 h at 4°C using a SW40 Ti rotor. Gradients were fractionated from top to bottom into a 96‐well plate and absorbance at 260 nm was measured using a microplate UV spectrophotometer (Ueta et al. 2017; Yoshida et al. 2021).

### 2.6 Area Under the Curve (AUC) Calculation

Ribosome sedimentation profiles were exported as numerical data for quantitative analysis. For each profile, a baseline was defined and the area under the curve (AUC) was calculated for user‐defined peak boundaries corresponding to each ribosome species. AUC values were computed by numerical integration using the trapezoidal rule across the selected interval, according to the following equation:

$$\mathrm{AUC}\left(\text{Area under the curve}\right)=\sum_{i=1}^{n-1}\frac{\left(y_{i}+y_{i+1}\right)}{2}\times \left(x_{i+1}-x_{i}\right)$$

where $x_{i}$ represents the fraction number and $y_{i}$ represents the corresponding absorbance at 260 nm (*A*_260_). Integration was performed across all data points within the user‐defined peak boundaries. Peak boundaries were kept identical across biological replicates within an experiment to ensure consistent quantification. To enable comparison across runs and to account for variations in total ribosome loading, AUC values were normalised to the total AUC of each profile. Relative ribosome abundances were reported as fractional AUC (AUC_peak_/AUC_total_) and as peak ratios calculated using normalised AUC values (e.g., 70S/(90–100S)). All values are presented as mean ± standard error of the mean (SEM) from independent biological replicates.

### 2.7 Statistical Analysis

**FIGURE 1:**
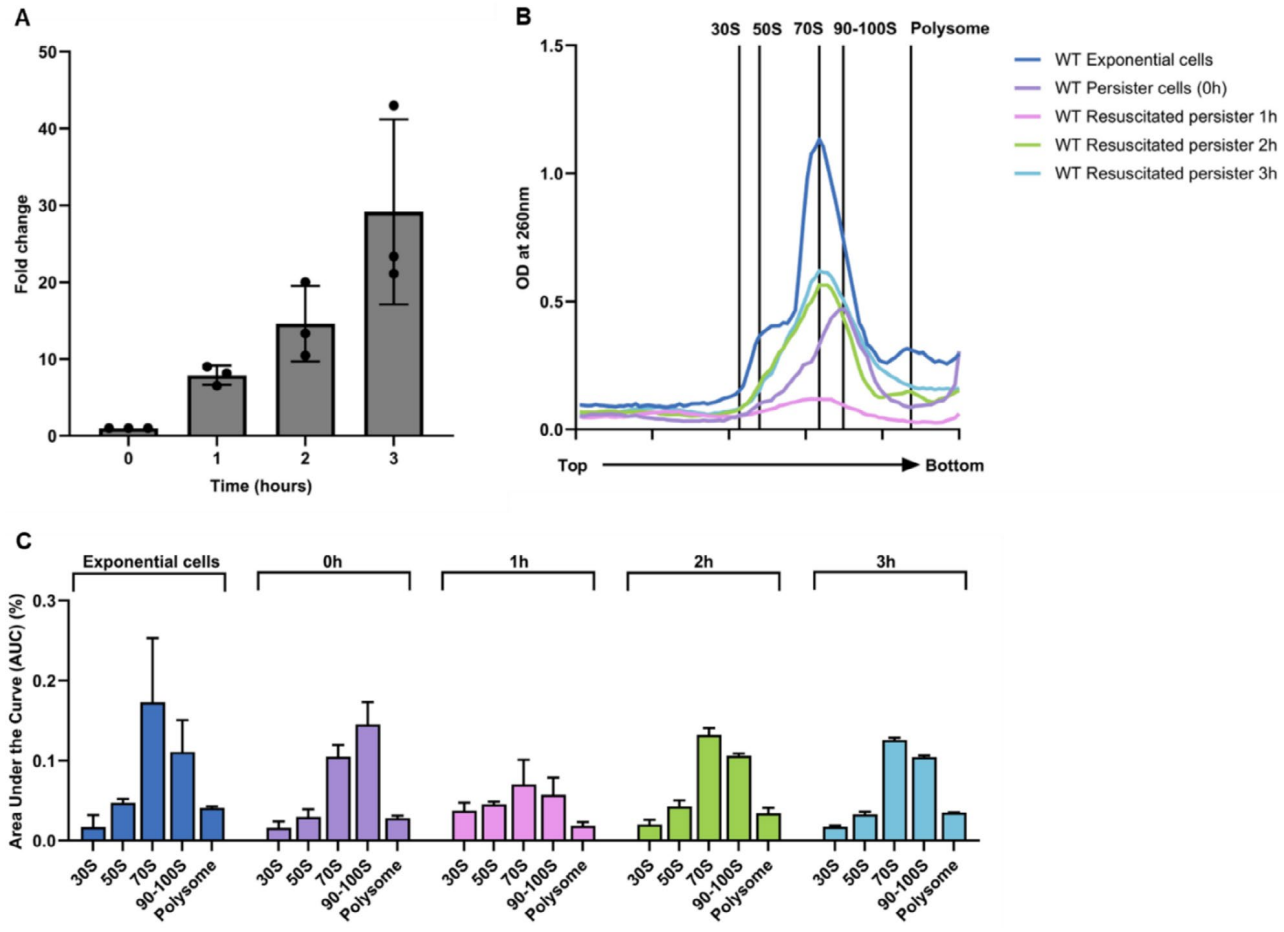
Ribosome sedimentation profiling of resuscitating persister cells. (A) Resuscitation kinetics of BW25113 WT persister cells. Resuscitation fold change of BW25113 WT rifampicin‐induced persister cells measured at 0, 1, 2 and 3 h after transfer to fresh medium. Values were normalised to viable cell counts at 0 h (set to 1). Data represent mean ± SEM from three independent experiments. (B) Ribosome sedimentation profiling during persister resuscitation. Ribosome sedimentation profiles of the same persister cells described in A. collected at 0, 1, 2 and 3 h during resuscitation. Ribosome sedimentation profiles of BW25113 WT exponential‐phase cells are shown for comparison. Crude ribosomes were separated on 5%–20% sucrose density gradients without glutaraldehyde stabilisation and monitored at OD_260_. Top and bottom indicate gradient positions. (C) Remodelling of ribosome composition during resuscitation. Quantification of ribosomal species by area under the curve (AUC) analysis of the profiles shown in B, comparing persister cells during resuscitation and exponential‐phase cells.

All quantitative data are presented as mean ± SEM. Statistical analyses and graphical visualisations were performed using GraphPad Prism version 10 (GraphPad Software, San Diego, CA, USA). All experiments were performed with a minimum of three independent biological replicates (*n* ≥ 3), as specified in the corresponding figure legends. For persister formation assays and AUC‐based ribosome distribution comparisons, statistical significance was assessed by two‐way analysis of variance (ANOVA) followed by Tukey's multiple comparisons post hoc test. For resuscitation kinetics (Figure 1A), fold‐change values were compared across time points using one‐way ANOVA with Dunnett's post hoc test relative to the 0 h condition. To evaluate the consistency of the AUC_70S/AUC_100S ratio as a quantitative marker for the 100S‐enriched ribosome state, statistical validation was performed across 84 independent populations (*N* = 84 total), including laboratory (BW25113) and clinical ( *E. coli* O157:H7) strains. An unpaired *t*‐test (two‐tailed) was employed to compare the distribution of ratios between non‐persister (*n* = 27) and persister‐state (*n* = 57) samples across all experimental conditions. Additionally, a point‐biserial correlation analysis was conducted to assess the association between the AUC_70S/AUC_100S ratio and the bacterial physiological state (non‐persister vs. persister). All statistical analyses and visualisations, including box plots representing the median and interquartile range (IQR), were performed using Python (SciPy and Seaborn libraries). A *P*‐value of less than 0.05 was considered statistically significant, with specific significance levels indicated in the figures (Figure S1).

Significance thresholds were defined as *p* ≤ 0.05 (*), *p* ≤ 0.01 (**) and *p* ≤ 0.001 (***). All AUC values derived from ribosome sedimentation profiles are reported as mean ± SEM. Raw numerical data underlying ribosome sedimentation profiles and quantitative analyses are available from the corresponding author upon reasonable request.

## 3 Results

### 3.1 Persister Cells Are Enriched for 90–100S Ribosome Complexes and Restore Growth‐Associated Ribosome Distributions During Resuscitation

100S ribosomes were shown for the first time to be enriched by 400% in persister cells generated with rifampicin compared to exponentially growing cells (Song and Wood 2020). To corroborate the increase in 100S ribosome population seen previously (Song and Wood 2020) as well as to examine the time course of 100S ribosome levels during persistence along with ribosome levels during resuscitation, we performed sucrose gradient‐based ribosome sedimentation profiling on *E. coli* persister cells generated by rifampicin pretreatment (Kwan et al. 2013) which has been rigorously validated to generate cells exhibiting the defining characteristics of true persisters, including multidrug tolerance without genetic change and full restoration of antibiotic susceptibility upon regrowth. This protocol has since been widely adopted in mechanistic studies of bacterial persistence, including those examining ribosome states and resuscitation dynamics (Yamasaki et al. 2020; Song and Wood 2020; Blattman et al. 2024). We then compared the ribosome sedimentation profiles of these persister cells with those of exponentially growing cells. WT persister cells displayed a prominent peak in the 90–100S range that was not prominent in exponential‐phase cells (Figure 1B).

To assess how ribosome complexes change during recovery, persister cells were transferred to fresh nutrient‐rich medium and samples were collected for ribosome sedimentation profiling at 1, 2 and 3 h post‐transfer (Figure 1B,C). Profile areas were quantified by integrating the area under the curve (AUC) for each defined region and expressing them as fractions of the total ribosome signal (AUC_Total). WT persister cells were characterised by a high 100S fraction (AUC_100S/AUC_Total) and comparatively low 70S and polysome fractions, indicating enrichment in translation‐repressed ribosome complexes (Figure 1C; Table S1). In contrast, exponentially growing cells exhibited higher 70S and polysome fractions and a reduced 100S fraction (Figure 1C; Table S1).

The relative balance between 70S and 100S complexes was summarised using the optional 70S‐to‐100S AUC ratio (AUC_70S/AUC_100S) (Table S1). This ratio was 1.5 in exponential‐phase cells but 0.7 in persister cells, indicating a shift from a 70S‐enriched (> 1) to a 100S‐enriched (< 1) distribution. Ratios > 1 are consistent with a more translation‐associated profile, whereas ratios < 1 are consistent with the translation‐repressed, persister‐like profile.

During resuscitation, ribosome‐complex distributions progressively shifted toward the exponential‐phase pattern. Within 1 h, the 100S fraction decreased while the 70S and polysome fractions increased (Figure 1C; Table S1). In parallel, viable counts increased by 7.9 ± 0.7‐fold at 1 h and further to 14.6 ± 2.8‐fold and 29.1 ± 7.0‐fold at 2 h and 3 h, respectively, relative to 0 h (Figure 1A; Table S2). By 3 h, the overall ribosome profile closely resembled that of exponentially growing cells (Figure 1B,C).

Collectively, these data indicate that rifampicin‐induced WT persister cells are enriched for 90–100S ribosome complexes and that resuscitation is accompanied by a progressive reduction of the 100S fraction with restoration of 70S ribosomes and polysomes, converging on a ribosome distribution characteristic of exponential growth.

### 3.2 RMF, RaiA, HPF and HflX Contribute to Persister Cell Formation and Resuscitation

To examine how ribosome‐hibernation factors shape ribosome‐complex distributions in persister cells, we performed sucrose gradient‐based ribosome sedimentation profiling on deletion mutants and compared their profiles with those of WT persister cells. All strains were harvested at 0.8 (OD_600_) in exponential phase prior to antibiotic exposure and persister formation (%) was quantified after treatment.

**FIGURE 2:**
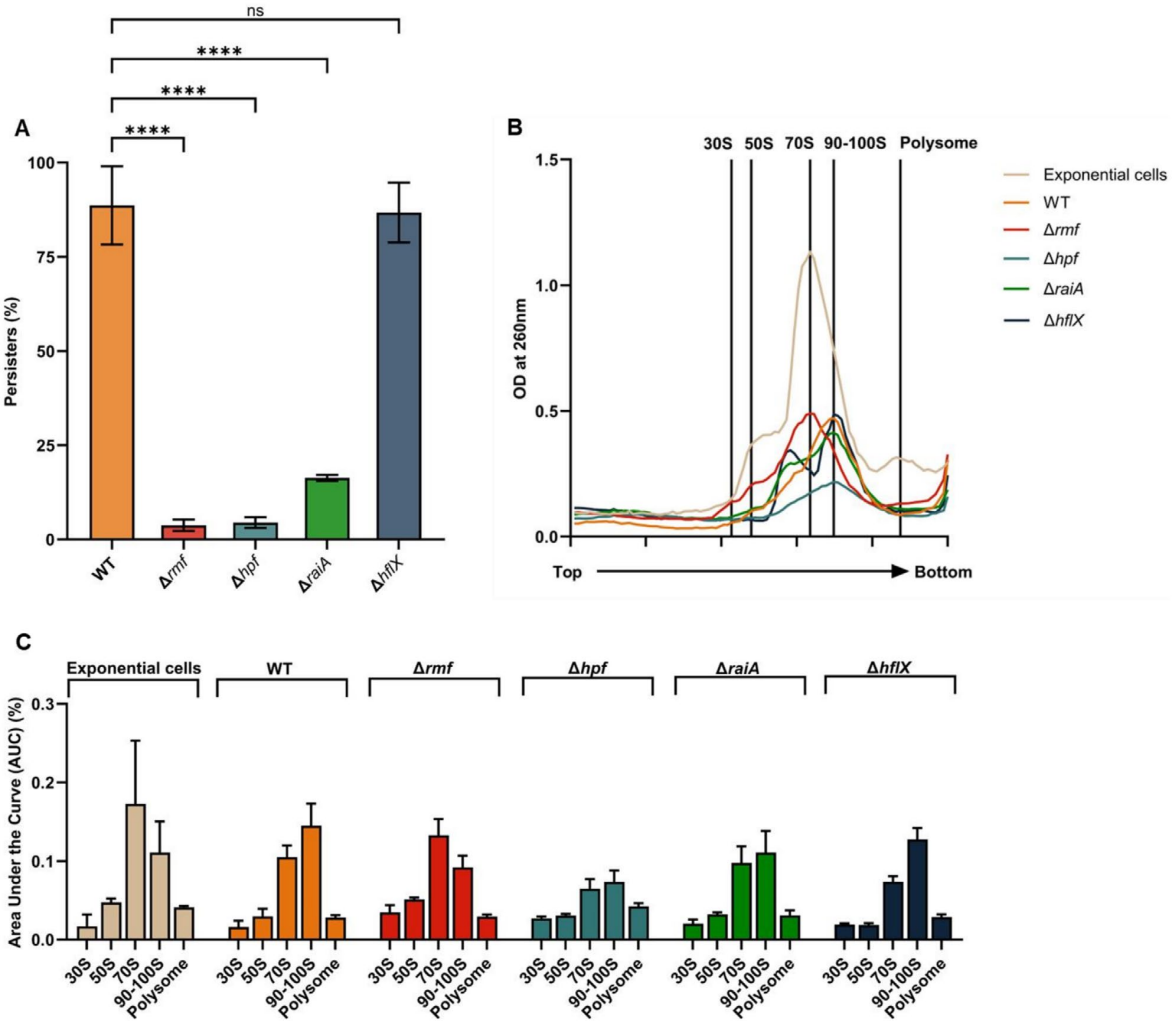
Ribosome sedimentation profiles and persister levels in ribosome‐associated factor mutants. (A) Persister levels in ribosome factor mutants. Persister levels of BW25113 WT, Δ*hpf*, Δ*rmf*, Δ*raiA* and Δ*hflX* strains following rifampicin treatment. Data represent mean ± SEM from independent experiments (*n* = 3 for BW25113 WT and Δ*hflX*; *n* = 6 for Δ*hpf*, Δ*rmf* and Δ*raiA*). Statistical differences were evaluated by two‐way ANOVA using GraphPad Prism 10; significance levels are indicated as *p* ≤ 0.0001 (***). (B) Ribosome sedimentation profiles of mutant persister cells. Ribosome sedimentation profiles of rifampicin‐induced persister cells from the strains shown in A. BW25113 WT exponential‐phase cells (OD_600_ = 0.8) are included for comparison. Crude ribosomes were separated on 5%–20% sucrose density gradients without glutaraldehyde stabilisation and monitored at OD_260_. Top and bottom indicate gradient positions. (C) AUC analysis of ribosome distribution in factor mutants. Quantification of ribosomal species by area under the curve (AUC) analysis of the profiles shown in B, comparing ribosome distributions among WT and mutant persister cells.

Consistent with the prior finding that *rmf* deletion abolishes 100S ribosome formation in persister cells (Song and Wood 2020), Δ*rmf* persister cells differed most from the WT persister ribosome sedimentation profile (high 90–100S fraction and lower 70S fraction) among the deletion strains examined. Δ*rmf* cells showed a decrease in the 90–100S fraction to 0.077 ± 0.009 and an increase in the 70S fraction to 0.105 ± 0.014, yielding a 70S‐to‐100S AUC ratio of 1.389 ± 0.127 (Figure 2C; Table S1). In parallel, persister formation decreased to 3.8% ± 0.9% of the population, corresponding to a 25.4 ± 4.1‐fold decrease relative to WT (Figure 2A; Table S3).

Deletion of *hpf* and *raiA* also decreased the 100S fraction compared with WT persister cells. The 70S‐to‐100S AUC ratios of Δ*hpf* and Δ*raiA* persister cells were 0.827 ± 0.046 and 0.758 ± 0.060, respectively (Figure 2C; Table S1). Persister formation decreased to 4.5% ± 0.8% in Δ*hpf* and 16.3% ± 0.5% in Δ*raiA*, corresponding to 20.8 ± 2.9‐fold and 5.5 ± 0.5‐fold decreases relative to WT, respectively (Figure 2A; Table S3).

Deletion of *hflX*, a ribosome rescue factor rather than a hibernation factor, did not affect persister formation relative to WT under the conditions tested. Δ*hflX* cells exhibited persister formation of 86.7% ± 4.6%, with a fold‐change of 1.0 ± 0.1 relative to WT (Figure 2A; Table S3). Ribosome sedimentation profiling of Δ*hflX* persister cells showed a 70S‐to‐100S AUC ratio of 0.579 ± 0.009, indicating retention of a 100S‐enriched ribosome distribution similar to WT persister cells (Figure 2C; Table S1).

**FIGURE 3:**
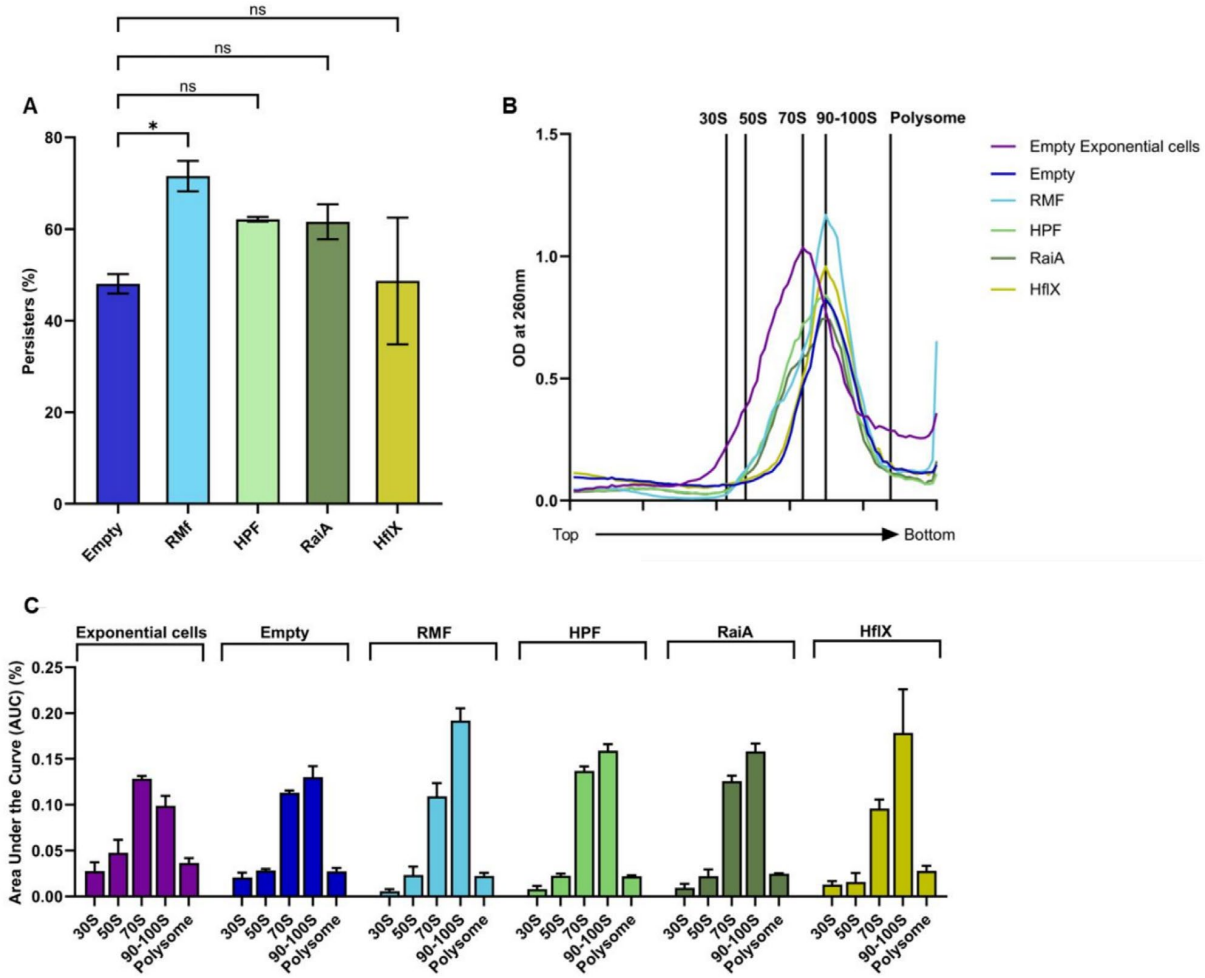
Ribosome sedimentation profiles and persister levels upon ribosome factor overexpression. (A) Persister levels upon ribosome factor overexpression. Persister levels of rifampicin‐induced cells carrying pCA24N‐empty, pCA24N‐*rmf*, pCA24N‐*hpf*, pCA24N‐*raiA* or pCA24N‐*hflX* plasmids. Data represent mean ± SEM from three independent experiments. Statistical significance was assessed by two‐way ANOVA using GraphPad Prism 10, with significance thresholds defined as *p* ≤ 0.05 (*). (B) Ribosome sedimentation profiles upon ribosome factor overexpression. Ribosome sedimentation profiles of rifampicin‐induced persister cells from the same strains described in A. Ribosome sedimentation profiles of pCA24N‐empty exponential‐phase cells are included for comparison. Crude ribosomes were separated on 5%–20% sucrose density gradients without glutaraldehyde stabilisation and monitored at OD_260_. Top and bottom indicate gradient positions. (C) AUC analysis of ribosome distribution upon ribosome factor overexpression. Quantification of ribosomal species by area under the curve (AUC) analysis of the profiles shown in B, comparing ribosome distributions between overexpression strains and the pCA24N‐empty control.

In overexpression experiments, ribosome sedimentation profiling was performed on persister cells carrying pCA24N‐based expression plasmids and compared with the empty‐vector control. The pCA24N‐empty strain exhibited a ribosome sedimentation profile consistent with WT cells, with persister cells showing elevated 90–100S fractions (0.130 ± 0.008) compared with exponential‐phase cells (0.099 ± 0.008) (Figure 3C; Tables S1 and S3).

Overexpression of RMF increased the 90–100S fraction to 0.192 ± 0.008 and decreased the 70S‐to‐100S AUC ratio to 0.572 ± 0.059 (Figure 3C; Tables S1 and S3). In parallel, persister formation increased to 71.6% ± 2.3%, corresponding to a 1.5 ± 0.1‐fold increase relative to the pCA24N‐empty control (Figure 3A; Table S3).

Overexpression of HPF and RaiA also increased the 100S fraction relative to the empty‐vector control (0.159 ± 0.005 and 0.158 ± 0.006, respectively) and maintained 70S‐to‐100S AUC ratios below 1 (Figure 3C; Table S1). Persister formation increased to 62.2% ± 0.3% and 61.6% ± 2.7% in the HPF‐ and RaiA‐overexpressing strains, corresponding to approximately 1.3‐fold increases relative to the control (Figure 3A; Table S3).

In contrast, overexpression of HflX did not change persister formation relative to the pCA24N‐empty control. Although the HflX‐overexpressing strain showed a 70S‐to‐100S AUC ratio of 0.579 ± 0.135 (Figure 3C; Tables S1 and S3), persister formation remained 48.6% ± 8.0%, with a fold‐change of 1.0 ± 0.2 relative to the control (Figure 3A; Table S3). Together, these results show that increased expression of RMF, HPF and RaiA is associated with an increased 100S fraction and increased persister formation, whereas HflX, a ribosome rescue factor, has minimal effects on persister formation under the conditions tested.

### 3.3 Stress‐Induced Persister Cells Exhibit Condition‐Dependent Formation but Shared 100S‐Enriched Ribosome Distributions

**FIGURE 4:**
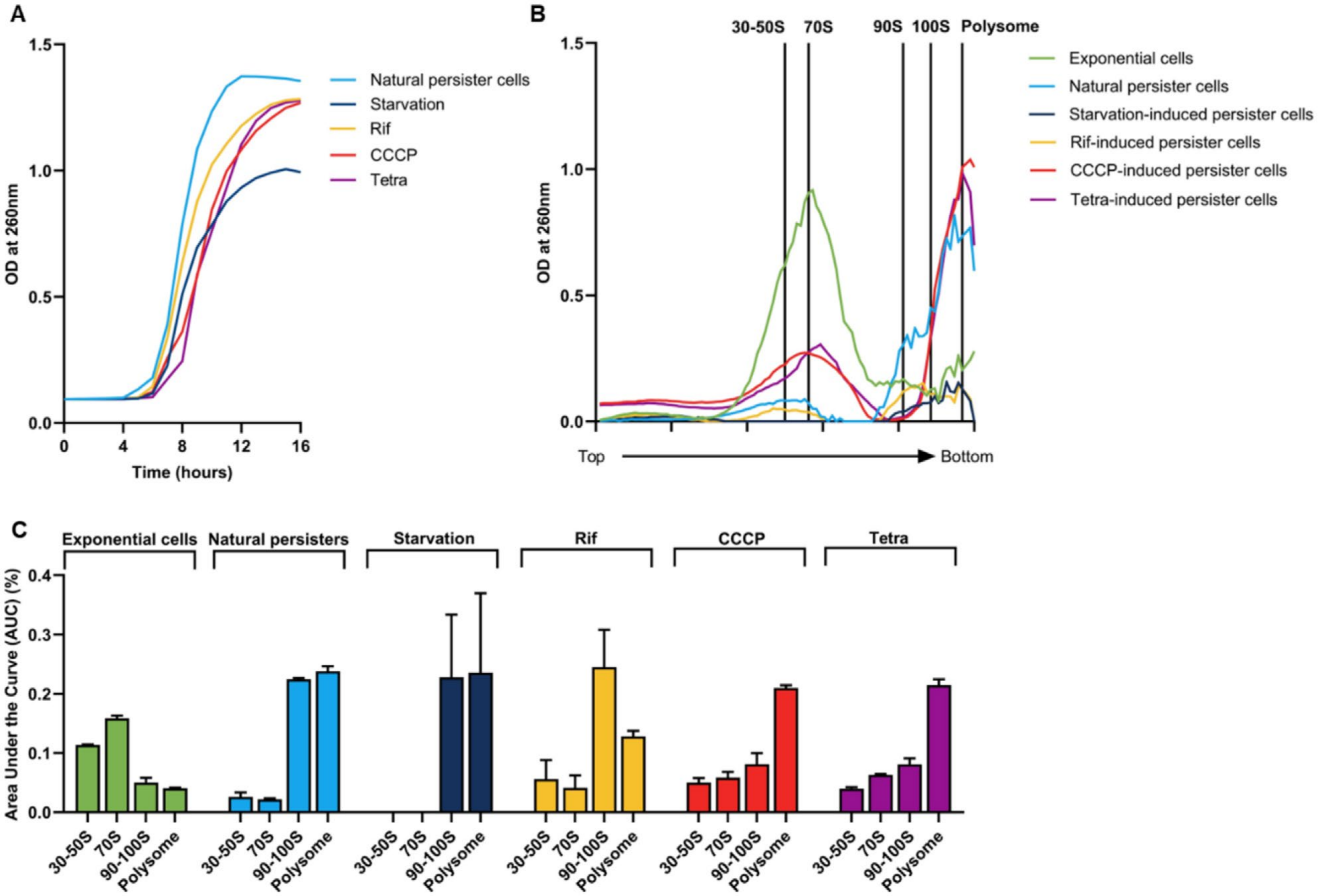
Ribosome sedimentation profiling and resuscitation dynamics of *E. coli* persister cells. (A) Resuscitation kinetics of diverse persister populations. Resuscitation kinetics of rifampicin‐, tetracycline‐ and CCCP‐induced persister cells, natural persister cells and 6‐day starved cells monitored for 16 h following transfer to fresh LB medium. Initial viable cell counts were normalised to 10^3^ CFU/mL and cultures were inoculated at 1% into LB broth. Growth recovery was assessed by measuring optical density (OD_600_) over time. (B) Ribosome sedimentation profiles of diverse persister populations. Ribosome sedimentation profiles of BW25113 WT exponential‐phase cells and the persister populations described in A. Samples were analysed on 5%–20% sucrose density gradients containing glutaraldehyde and monitored at OD_260_. (C) AUC analysis of ribosome distribution across persister populations. Quantification of ribosomal species by area under the curve (AUC) analysis of the profiles shown in B, comparing ribosome distributions among the indicated persister populations.

To determine whether persister populations generated by different stresses share common ribosome‐complex features and recovery behaviours, we compared persister formation, ribosome‐complex distributions and resuscitation across multiple induction conditions. For antibiotic‐induced persister cells and natural persister cells, cells were harvested at the exponential phase (OD_600_ = 0.8) prior to antibiotic exposure, whereas starvation‐induced persister cells were collected in the stationary phase following prolonged nutrient deprivation (Table S4). Persister formation was quantified after antibiotic treatment. Ribosome‐complex distributions were analysed by ribosome sedimentation profiling using sucrose gradients prepared with two compositions (−G or + G) (Figure 4B; Figure S2A; Table S5). Because CCCP‐induced persister cells showed a redistribution of ribosome signal toward lighter fractions under the −G condition, we used the +G condition to ensure consistent resolution of the 90–100S region for cross‐condition comparisons. Resuscitation was monitored after nutrient replenishment by measuring optical density over time (Figure 4A).

Persister formation differed across conditions (Table S4). Natural persister cells were detected at 0.003% ± 0.0001% of persister formation, whereas starvation‐induced persister formation was 0.6% ± 0.2%. Antibiotic‐based induction produced higher persister formation, with rifampicin‐induced persister cells comprising 5.0% ± 1.2% of the population and CCCP‐induced persister cells 18.6% ± 1.1%. Tetracycline‐induced persister cells showed the highest persister formation, at 96.8% ± 11.9%.

Using the +G condition, all stress‐induced persister populations, including starvation‐induced persister cells collected in the stationary phase, showed increased 90–100S fractions and 70S‐to‐100S AUC ratios (AUC_70S/AUC_100S) below 1 compared with exponential‐phase cells (Figure 4B; Table S5). This indicates a 100S‐enriched ribosome distribution across diverse induction methods.

Finally, we monitored recovery after nutrient replenishment by tracking optical density over time (Figure 4A). All persister populations resumed growth after nutrient replenishment (Figure 4A). Quantitatively, t_OD_600_ = 1.0 was 9 h (natural), 14 h (starvation), 10 h (rifampicin), 11 h (CCCP) and 12 h (tetracycline). Together, these data show that persister cells generated by diverse stresses share a common feature of 100S‐enriched ribosome distributions, while resuscitation timing differs across induction conditions.

### 3.4 Conserved Ribosome State Remodelling and Resuscitation Dynamics in a Clinically Relevant *E. coli* O157:H7 Strain

**FIGURE 5:**
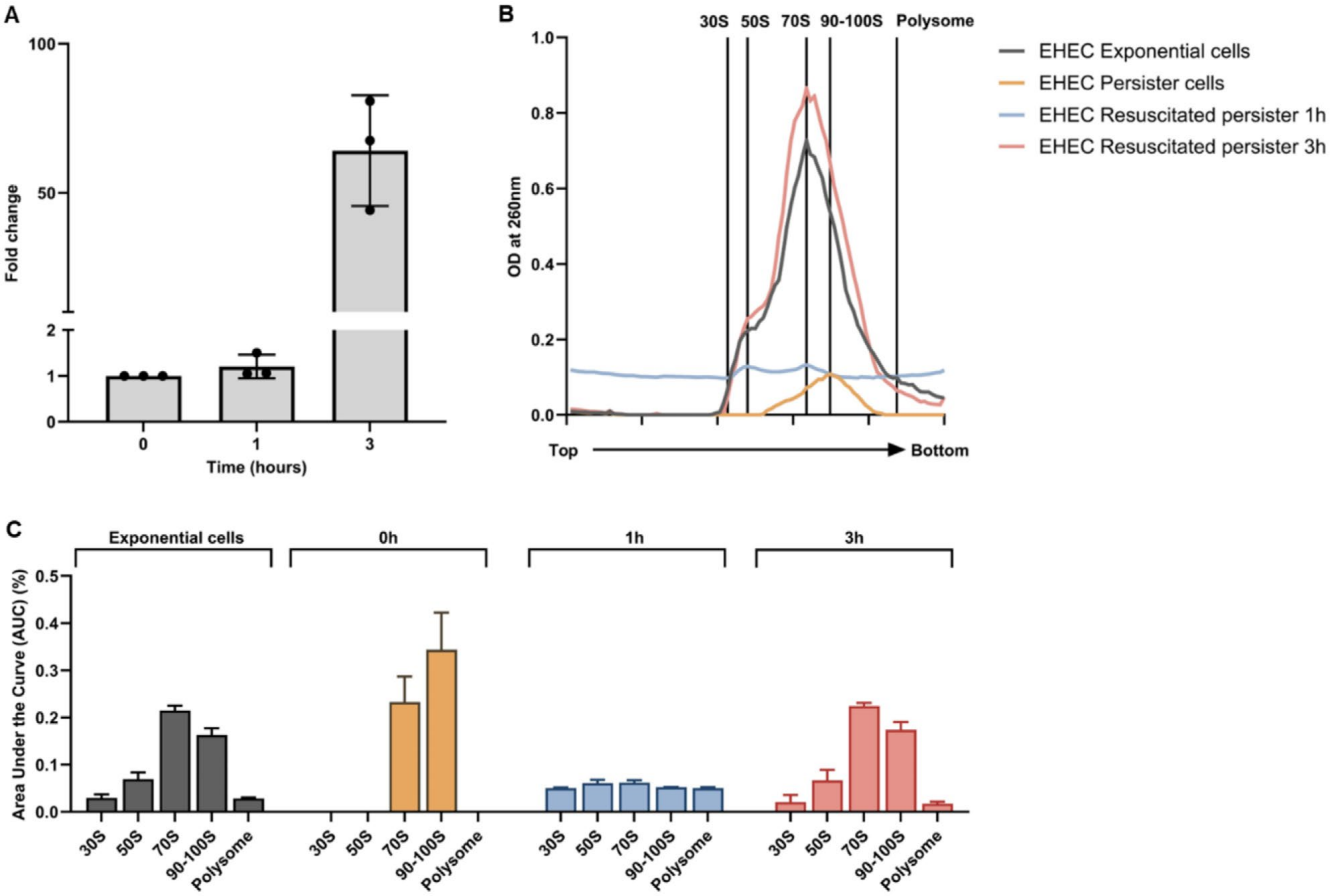
Ribosome sedimentation profiling of resuscitating clinical *E. coli* O157:H7 persister cells. (A) Resuscitation kinetics of EHEC persister cells. Resuscitation fold change of EHEC natural persister cells measured at 0, 1 and 3 h after transfer to fresh medium. Values were normalised to viable cell counts at 0 h (set to 1). Data represent mean ± SEM from three independent experiments. (B) Ribosome sedimentation profiling during EHEC persister resuscitation. Ribosome sedimentation profiles of the same persister cells described in A. collected at 0, 1 and 3 h during resuscitation. Crude ribosomes were separated on 5%–20% sucrose density gradients without glutaraldehyde stabilisation and monitored at OD_260_. Top and bottom indicate gradient positions. (C) Remodelling of ribosome composition during EHEC resuscitation. Quantification of ribosomal species by area under the curve (AUC) analysis of the profiles shown in B, comparing EHEC persister cells during resuscitation.

To determine whether the ribosome‐centric mechanisms of persistence are conserved beyond BW25113 strains, we investigated a clinically relevant pathogen, enterohemorrhagic *E. coli* (EHEC) O157:H7. We performed ribosome sedimentation profiling on natural EHEC persister populations and monitored their resuscitation dynamics (Figure 5). Similar to the BW25113 strain, EHEC persister cells (0 h) exhibited a marked suppression of 70S ribosomes and a pronounced enrichment in 90–100S inactive assemblies (Figure 5B).

Consistent with our quantitative analysis of the laboratory strain, the AUC_70S/AUC_100S ratio effectively captured this structural shift in EHEC (Figure 5C, Table S6). The ratio was approximately 1.3 in EHEC exponential‐phase cells but dropped to 0.7 in persister cells, confirming the 100S‐enriched state. During resuscitation, this ratio progressively recovered: it increased to 1.2 within 1 h post‐transfer and reached 1.3 by 3 h, driven by the massive restoration of 70S ribosomes.

In parallel with these ribosome state transitions, EHEC cells demonstrated progressive growth recovery upon nutrient replenishment. The initial remodelling of the ribosome pool at 1 h preceded overt population growth, while the profound 70S restoration at 3 h coincided with an exponential increase in viable cell counts (Figure 5A). Taken together, these data indicate that the temporal remodelling of the ribosome pool extends beyond laboratory models. The strong parallel between the clinically relevant EHEC strain and the BW25113 strain—both exhibiting a consistent quantitative reversal from a 100S‐enriched hibernation state (Ratio < 1.0) to a 70S‐restored active state (Ratio > 1.0) preceding overt growth—supports the notion that this structural transition is a shared physiological feature of *E. coli* persistence. To rigorously validate the robustness of this quantitative shift, we next performed a comprehensive statistical analysis integrating all experimental conditions and strains examined in this study.

### 3.5 Statistical Analysis of the AUC_70S/AUC_100S Ratio Across Diverse Conditions

To evaluate the consistency of the AUC_70S/AUC_100S ratio as a quantitative marker for the persister ribosome state, we performed an integrative statistical analysis across 84 independent populations examined in this study (Figure S1; Table S7). This extensive dataset included various stress‐induced populations and clinical *E. coli* O157:H7 (EHEC) strains. Overall, the AUC_70S/AUC_100S ratio was significantly lower in persister‐state samples compared to non‐persister‐state samples (0.709 ± 0.050 vs. 1.500 ± 0.125; *p* < 0.0001, unpaired *t*‐test). Furthermore, a point‐biserial correlation analysis revealed a strong, statistically significant negative association between the AUC_70S/AUC_100S ratio and the persister state (r = −0.603, *p* < 0.0001; Figure S1B).

While a threshold of AUC_70S/AUC_100S < 1.0 was observed across all wild‐type persister‐state samples, the Δ*rmf* mutant (ratio = 1.389) was a notable biological outlier. This finding is consistent with the severely attenuated persister formation in this mutant (3.8% relative to WT) and reflects its inability to establish a canonical 100S‐enriched ribosome distribution.

Taken together, these statistical results demonstrate that a shift in the AUC_70S/AUC_100S ratio to below 1.0 is consistently observed across various *E. coli* persister populations induced by different stresses. This quantitative transition reflects a significant and reproducible reorganisation of the ribosome pool toward non‐productive states during the establishment of the persister state under the conditions tested.

## 4 Discussion

Bacterial persister states may be more informatively characterised by their ribosome state distribution, than by a generalised cessation of growth or metabolism (Song and Wood 2020; Maki and Yoshida 2021). In rifampicin‐induced *E. coli* persister cells, sedimentation profiling revealed a 100S‐enriched ribosome distribution, marked by an elevated 90–100S fraction and 70S reduction, clearly distinguishing persister cells from exponentially growing cells (Figure 1B,C; Table S1). These observations are consistent with the ppGpp ribosome dimerisation persister (PRDP) model and support a quantitative link between ribosome‐complex distribution metrics (e.g., AUC_100S/AUC_Total and AUC_70S/AUC_100S) and persister formation (Song and Wood 2020).

Exit from persistence was accompanied by ordered remodelling of ribosome state distributions. Within 1 h of resuscitation, the 100S fraction declined, whereas the 70S and polysome fractions increased (Figure 1C; Table S1), concomitant with an increase in the number of cells exiting the persister state (Figure 1A; Table S2). Consistent with this shift, the 70S‐to‐100S AUC ratio (AUC_70S/AUC_100S) increased from a persister‐like, 100S‐enriched state (< 1) to a more translation‐associated state (> 1) within the first hour (Table S1). By 3 h, polysome fractions approached exponential‐phase levels (Figure 1C), indicating that restoration of growth‐associated ribosome distributions follows an early shift away from 100S enrichment. This interpretation is mechanistically consistent with previous work showing that the conserved GTPase HflX can dissociate hibernating 100S ribosomes, thereby facilitating their return to translation‐competent states (Basu and Yap 2017). Together, this temporal pattern suggests that remodelling of ribosome state distributions occurs in parallel with growth recovery and may represent a required step preceding measurable resuscitation. This interpretation is also consistent with previous reports showing that persister waking involves early ribosome reactivation and restoration of translation‐associated machinery before overt growth resumes (Kim, Yamasaki, et al. 2018; Song and Wood 2020; Semanjski et al. 2021).

The replication of these ribosome dynamics in the pathogenic EHEC strain demonstrates that 100S‐mediated hibernation is a conserved physiological feature of the *E. coli* persister state. The consistent quantitative shift observed across both laboratory and pathogenic strains—characterised by a 100S‐enriched hibernation state (Ratio < 1.0) transitioning to a 70S‐restored active state (Ratio > 1.0)—supports the notion that this structural remodelling is a shared characteristic of *E. coli* persistence, regardless of the genetic background.

Gene‐deletion analysis further clarified the relationship between ribosome‐state distribution and persister formation. Consistent with the previous demonstration that *rmf* deletion abolishes 100S ribosome formation in persister cells (Song and Wood 2020), deletion of *rmf* produced the most distinct ribosome sedimentation profile relative to WT persister cells, markedly reducing the 90–100S fraction and shifting the 70S‐to‐100S AUC ratio above 1 (Figure 2C; Table S1), accompanied by a sharp decrease in persister formation (Figure 2A; Table S3). The increased 70S fraction in Δ*rmf* cells is consistent with accumulation of inactive 70S ribosomes, potentially including RaiA‐bound monomers, whereas the reduced residual 100S fraction may reflect incomplete or destabilised hibernating complexes relative to the mature RMF/HPF‐dependent 100S population in WT persister cells. This interpretation is supported by previous studies showing that RMF initiates 90S/100S assembly, HPF stabilises the mature 100S complex and RaiA preferentially associates with inactive 70S monomers and can antagonise RMF‐dependent 100S formation (Ueta et al. 2005; Beckert et al. 2018; Maki and Yoshida 2021). Deletion of *hpf* or *raiA* similarly reduced the 100S fraction and altered the AUC ratio relative to WT persister cells (Figure 2C; Table S1) and these changes were also associated with reduced persister formation (Figure 2A; Table S3). In contrast, deletion of *hflX* had little effect on persister formation (Figure 2A; Table S3) and retained a 100S‐enriched, persister‐like ribosome state distribution (Figure 2C; Table S1), suggesting that HflX is dispensable for establishing the 100S‐enriched persister state under these conditions.

Overexpression analysis supported these conclusions. Increased expression of RMF, HPF or RaiA enhanced the 90–100S fraction and maintained AUC_70S/AUC_100S below 1 (Figure 3C; Table S1), accompanied by increased persister formation (Figure 3A; Table S3). In contrast, HflX overexpression altered ribosome‐complex distributions but did not reduce persister formation (Figure 3A,C; Tables S1 and S3). Together, these results suggest that RMF plays a dominant role in establishing the 100S‐enriched ribosome distribution characteristic of rifampicin‐induced persister cells, whereas HPF and RaiA contribute to its maintenance, consistent with previous studies showing that RMF initiates 100S assembly, HPF stabilises mature 100S complexes and RaiA is associated primarily with inactive 70S monomers rather than canonical 100S dimers (Ueta et al. 2005; Beckert et al. 2018; Maki and Yoshida 2021). By contrast, HflX does not measurably affect persister formation under the conditions tested in this study, consistent with its demonstrated role in resuscitation, where HflX dissociates hibernating 100S ribosomes into active 70S monomers and *hflX* deletion completely abolishes persister resuscitation (Yamasaki et al. 2020).

Across stress conditions, translational arrest was associated with distinct ribosome state distributions. Starvation‐ and rifampicin‐induced persister cells exhibited organised 100S accumulation under stabilised conditions (Figure 4B; Table S5) and natural persister cells selected by ampicillin displayed comparable profiles (Figure 4B; Table S5). In contrast, CCCP‐induced persister cells, formed following abrupt collapse of cellular energy production (Le et al. 2021) and tetracycline‐induced persister cells, generated through immediate inhibition of translation elongation (Chopra and Roberts 2001), displayed ribosome state distributions distinct from those of starvation‐ and rifampicin‐induced cells (Figure 4B; Table S5). Notably, both CCCP‐ and tetracycline‐induced persister cells exhibited elevated polysome‐like peaks relative to exponential‐phase cells; however, their delayed recovery (Figure 4A) suggests that these signals do not represent productive translation. Despite these structural variations, the AUC_70S/AUC_100S ratio consistently remained below 1.0 across all examined stressors, indicating a shared quantitative shift in the ribosome pool balance (Figure 4C; Table S5). Instead, these peaks likely reflect stalled or otherwise non‐productive ribosome assemblies that accumulate when cellular energy collapses or elongation is abruptly arrested. It should be noted that the present study does not directly measure translational activity within each ribosomal fraction, a limitation that applies to two key aspects of our findings. First, while our interpretation of 100S ribosomes as translationally inactive complexes is well‐supported by established structural and biochemical evidence (Kato et al. 2010; Polikanov et al. 2012), direct confirmation under specific persister conditions remains for future investigation. Second, we infer that the polysome‐like peaks observed in CCCP‐ and tetracycline‐induced persisters reflect non‐productive ribosome assemblies rather than active translation. This inference is based on two lines of evidence: (i) tetracycline is known to trap ribosomes in a non‐productive state on mRNA by blocking aminoacyl‐tRNA accommodation (Chopra and Roberts 2001) and (ii) CCCP abruptly collapses the proton motive force, creating cellular conditions incompatible with the high energy demands of translation (Le et al. 2021). Furthermore, the markedly delayed resuscitation kinetics observed in these populations (Figure 4A) support the interpretation that these assemblies are not translationally productive. Nevertheless, future studies employing Ribo‐seq or [^35^S]‐methionine pulse‐labelling will be required to formally validate the translational status of these ribosome fractions under diverse persister‐induction conditions. Despite these condition‐dependent differences, all persister populations showed a common shift away from the exponential‐phase ribosome profile, characterised by reduced 70S fractions, increased 90–100S fractions and elevated polysome‐like peaks (Figure 4B; Figure S2A; Table S5). These shared features indicate that functional ribosome distributions are broadly reorganised across induction conditions, although the resulting ribosome states differ in their structural and functional properties (Song and Wood 2020; Maki and Yoshida 2021). The subsequent resuscitation profiles further distinguished these states, as persister cells exhibiting organised 100S enrichment recovered more rapidly than those formed under abrupt energetic collapse or direct elongation inhibition (Figure 4A), suggesting that the route into persistence is linked to subsequent recovery dynamics (Kim, Yamasaki, et al. 2018; Semanjski et al. 2021).

Taken together, these findings support a ribosome‐centred view of persistence in which persister states are unified by suppression of translation‐associated ribosome distributions but diversified by condition‐dependent ribosome state distributions enriched in the 90–100S region. Although ribosome sedimentation profiling captures population‐level snapshots rather than single‐cell resolution, quantitative analysis of ribosome fractions and AUC‐derived metrics provides a useful mechanistic framework for interpreting heterogeneous persister behaviours across stresses and for linking ribosome state distributions to both persister formation and resuscitation dynamics. Although persister cells are known to undergo rRNA degradation under certain conditions (Cho et al. 2015), RNA integrity was verified by A260/A280 ratio measurements prior to analysis and equal RNA concentrations were applied consistently across all conditions following previously established normalisation approaches (Kato et al. 2010; Ueta et al. 2017). Crucially, the integrity of the ribosomal samples was further validated by the sedimentation profiles themselves; the absence of significant absorbance in the soluble (SOL) fractions at the top of the gradients, combined with the presence of sharp, well‐resolved ribosomal peaks, indicates that the observed distributions were not artefacts of rRNA degradation (Maguire et al. 2008). From a biotechnological perspective, the present findings carry several practical implications. First, the AUC_70S/AUC_100S ratio introduced here provides a simple, quantitative framework to assess the persister ribosome state. Our integrative analysis demonstrated a robust and statistically significant negative association between this ratio and the persister state across diverse experimental conditions (*r* = −0.603, *p* < 0.0001, *N* = 84), suggesting that this index could be integrated into screening workflows for anti‐persistence compounds (Figure S1; Table S7). Although a formal evaluation of sensitivity and specificity was not conducted in the present study, the consistent quantitative shift observed across 84 independent populations provides a foundational framework for such diagnostic validation (Table S7). While our current results are based on *E. coli* models, future studies employing even larger datasets spanning additional bacterial species and clinical contexts may enable formal receiver operating characteristic (ROC)‐based validation. Such efforts could further establish the utility of this ratio as a reliable indicator for monitoring the physiological transitions associated with bacterial persistence.

Agents that prevent or reverse 100S ribosome accumulation—for example by inhibiting RMF or HPF activity—may sensitise persister populations to antibiotic killing, offering a complementary strategy to conventional bactericidal treatment. Second, the observation that RMF deletion severely attenuates persister formation across induction conditions identifies RMF as a high‐priority molecular target, as previously demonstrated under ampicillin and ciprofloxacin conditions (Song and Wood 2020), consistent with recent interest in hibernation factors as candidates for therapeutic intervention (Maki and Yoshida 2021). Third, the cross‐condition convergence of ribosome state distributions on a shared 100S‐enriched profile suggests that ribosome sedimentation profiling may serve as a generalizable diagnostic tool for detecting persister physiology in diverse bacterial species and infection contexts. Collectively, these data advance understanding of persister biology and provide a quantitative ribosome‐state framework with direct relevance to the design of persister‐directed antimicrobial strategies.

## Author Contributions

S.S. conceived and designed the study, supervised the project, analysed and interpreted the data and wrote the manuscript. H.K. performed the experiments, contributed to data analysis and interpretation and co‐wrote the manuscript. All authors read and approved the final manuscript.

## Funding

This work was supported by the National Research Foundation of Korea (2020R1F1A1072397, RS‐2023‐00210305).

## Conflicts of Interest

The authors declare no conflicts of interest.

## Supporting information


**Table S1:** Ribosome state distribution quantified by ribosome sedimentation profiling in *E. coli*. Relative distributions of ribosomal species were determined by calculating the area under the curve (AUC) for each peak and normalising to the total AUC of the profile. “Total” represents the integrated area of the entire ribosome sedimentation profile and 30S, 50S, 70S, 90–100S and polysome correspond to the AUC of each respective peak. WT persister cells were generated by growing cultures in LB to OD_600_ = 0.8, followed by rifampicin treatment (30 min), ampicillin treatment (3 h) and washing with 0.85% NaCl. Gene deletion mutants (Δ*rmf*, Δ*hpf*, Δ*raiA* and Δ*hflX*; grown in LB supplemented with kanamycin) and plasmid‐bearing strains (pCA24N‐empty or gene‐expressing constructs; grown in LB supplemented with chloramphenicol) were processed identically to obtain persister cells. WT persister cells are designated as 0 h and serve as the reference point for subsequent resuscitation time points (1, 2 and 3 h). Values are expressed as mean ± SEM and rounded to three decimal places.
**Table S2:** Time‐dependent increase in persister cell resuscitation upon nutrient supply. Viable cell counts and resuscitation of rifampicin‐induced persister cells at 0 h and after 1–3 h of resuscitation. Persister cells (0 h) were used as the reference for fold‐change calculations. Values are presented as mean ± SEM. Fold‐change was calculated for each replicate relative to 0 h and then averaged.
**Table S3:** RMF, Hpf and RaiA promote persister formation. Viable cell counts of exponential‐phase cells at OD_600_ = 0.8 prior to antibiotic treatment and persister levels formed after antibiotic exposure in *E. coli* BW25113 and its derivatives. Values are presented as mean ± SEM. For gene deletion mutants (Δ*rmf*, Δ*hpf*, Δ*raiA* and Δ*hflX*), fold‐change was calculated for each replicate based on persister rates (%) relative to the BW25113 WT control and then averaged. For plasmid‐based overexpression strains, fold‐change was calculated for each replicate based on persister rates (%) relative to the BW25113 pCA24N‐empty control and then averaged. 70S‐to‐100S AUC ratios were calculated from ribosome sedimentation profiles obtained using sucrose density gradients without glutaraldehyde (−G).
**Table S4:** Distinct levels of persister formation under different inducing conditions. Viable cell counts and persister levels at 0 h in exponential cells and persister populations generated under different inducing conditions. Values are presented as mean ± SEM. Persister rates (%) were calculated as the percentage of persister CFU relative to total viable CFU. Rif, rifampicin; Tetra, tetracycline. 70S‐to‐100S AUC ratios were calculated from ribosome sedimentation profiles obtained using glutaraldehyde‐fixed (+G) sucrose density gradients.
**Table S5:** Ribosome sedimentation profiling–based analysis of ribosome states in persister cells induced by different stresses. Relative distributions of ribosomal species were determined by calculating the area under the curve (AUC) for each peak and normalising to the total AUC of the profile. “Total” represents the integrated area of the entire ribosome sedimentation profile and 30S, 50S, 70S, 90–100S and polysome correspond to the AUC of each respective peak. Persister cells were induced using rifampicin–ampicillin (Rif‐induced persister cells), carbonyl cyanide m‐chlorophenyl hydrazone–ampicillin (CCCP‐induced persister cells), tetracycline–ampicillin (Tetra‐induced persister cells) or prolonged starvation for 6 days (Starvation‐induced persister cells), as indicated. Ribosome sedimentation profiling was performed using sucrose density gradients prepared either without glutaraldehyde (−G) or with glutaraldehyde fixation (+G). Ribosome sedimentation profiling data were quantified using the area under the curve (AUC) method and values are expressed as ratios relative to the total ribosome signal. Values are expressed as mean ± SEM. All values are rounded to three decimal places.
**Table S6:** Quantitative analysis of ribosome state distribution and time‐dependent resuscitation of natural persister cells in *E. coli* EHEC O157:H7. Relative distributions of ribosomal species were determined by calculating the area under the curve (AUC) for each peak obtained from ribosome sedimentation profiling and normalising to the total AUC of the profile. “Total” represents the integrated area of the entire ribosome sedimentation profile and 30S, 50S, 70S, 90–100S and polysome correspond to the AUC of each respective peak. WT persister cells were generated by growing cultures in LB to OD_600_ = 0.8, followed by ampicillin treatment (3 h) and washing with 0.85% NaCl. WT persister cells are designated as 0 h and serve as the reference point for subsequent resuscitation time points (1 and 3 h). Viable cell counts and resuscitation of natural persister cells at 0 h and after 1 and 3 h of resuscitation were analysed. Persister cells (0 h) were used as the reference for fold‐change calculations. Fold‐change was calculated for each replicate relative to 0 h and then averaged. Values are presented as mean ± SEM and rounded to three decimal places.
**Table S7:** Statistical validation of the AUC_70S/AUC_100S ratio as a quantitative index across diverse *E. coli* populations. Individual AUC_70S/AUC_100S ratios from all experimental conditions examined in this study (*N* = 87 total) are listed alongside their corresponding state assignments (non‐persister vs. persister). State assignments were determined by experimental context: exponential‐phase cells and resuscitated cells were designated as non‐persister‐state (*n* = 30); all antibiotic‐ or stress‐induced populations were designated as persister‐state (*n* = 57). Ratios were obtained from ribosome sedimentation profiles using both laboratory (BW25113) and clinical ( *E. coli* O157:H7) strains. Statistical analysis using an unpaired *t*‐test (two‐tailed) confirmed a highly significant difference between the two groups (persister‐state: 0.709 ± 0.375 vs. non‐persister‐state: 1.488 ± 0.619, *p* < 0.0001). Furthermore, a point‐biserial correlation confirmed a strong and significant negative association between the AUC_70S/AUC_100S ratio and the persister state (*r* = −0.621, *p* < 0.0001). The single biological outlier, Δ*rmf* persister cells (ratio = 1.389), is consistent with the inability of this mutant to form canonical 100S‐enriched ribosome states under the tested conditions. All underlying data are consistent with the distributions shown in Figure S1.
**Figure S1:** Statistical analysis of the AUC_70S/AUC_100S ratio across diverse *E. coli* populations. The AUC_70S/AUC_100S ratio was analysed to evaluate its consistency as a quantitative marker for the 100S‐enriched ribosome state. (A) Distribution of AUC_70S/AUC_100S ratios in non‐persister (*n* = 27) and persister‐state (*n* = 57) samples across all experimental conditions, including laboratory (BW25113) and clinical ( *E. coli* O157:H7) strains. Box plots represent the median and interquartile range (IQR), with whiskers extending to 1.5 x IQR. Individual data points are shown as black circles (*N* = 84 total). Persister‐state samples exhibited significantly lower ratios compared to non‐persister samples (*p* < 0.0001), unpaired *t*‐test (two‐tailed). (B) Point‐biserial correlation analysis between the AUC_70S/AUC_100S ratio and the bacterial state (non‐persister vs. persister). A statistically significant negative association was confirmed (*r* = −0.603, *p* < 0.0001). *rmf* mutant persister samples (red circle; #1 ratio = 1.411, #2 ratio = 1.916, #3 ratio = 1.112, #4 ratio = 1.125, #5 ratio = 1.206 and #6 ratio = 1.561) are indicated as a biological outlier, consistent with its inability to form canonical 100S ribosomes under the conditions tested. All underlying data are provided in Supplementary Table S7.
**Figure S2:** Effect of glutaraldehyde stabilisation on ribosome sedimentation profiles of diverse *E. coli* persister populations. (A) Ribosome sedimentation profiles diverse persister populations without glutaraldehyde stabilisation. Ribosome sedimentation profiles of BW25113 WT exponential‐phase cells and the diverse persister populations described in Figure 4A. The same samples used in Figure 4 were analysed on 5%–20% sucrose density gradients without glutaraldehyde stabilisation and monitored at OD_260_. (B) AUC analysis of ribosome distributions without glutaraldehyde stabilisation. Quantification of ribosomal species by area under the curve (AUC) analysis of the profiles shown in A, comparing ribosome distributions among the indicated persister populations under non‐stabilised conditions.

## Data Availability

The data that support the findings of this study are available from the corresponding author upon reasonable request. Raw numerical data underlying ribosome sedimentation profiles, AUC calculations and persister‐formation assays are provided in the Supporting Information Tables (S1–S7). No large‐scale sequencing or omics datasets were generated in this study.
