# Supplementary material for: Ribosome State Distributions Define Escherichia coli Persister Physiology: Links to Formation, Stress Responses, and Resuscitation Dynamics
Source: Microb Biotechnol. 2026 Apr 13;19(4):e70352. doi: 10.1111/1751-7915.70352 (PMC13076356; doi:10.1111/1751-7915.70352)
Supplement: Supplementary file 1 — Table S1: Ribosome state distribution quantified by ribosome sedimentation profiling in E. coli. Relative distributions of ribosomal species were determined by calculating the area under the curve (AUC) for each peak and normalising to the total AUC of the profile. “Total” represents the integrated area of the entire ribosome sedimentation profile and 30S, 50S, 70S, 90–100S and polysome correspond to the AUC of each respective peak. WT persister cells were generated by growing cultures in LB to OD600 = 0.8, followed by rifampicin treatment (30 min), ampicillin treatment (3 h) and washing with 0.85% NaCl. Gene deletion mutants (Δrmf, Δhpf, ΔraiA and ΔhflX; grown in LB supplemented with kanamycin) and plasmid‐bearing strains (pCA24N‐empty or gene‐expressing constructs; grown in LB supplemented with chloramphenicol) were processed identically to obtain persister cells. WT persister cells are designated as 0 h and serve as the reference point for subsequent resuscitation time points (1, 2 and 3 h). Values are expressed as mean ± SEM and rounded to three decimal places. Table S2: Time‐dependent increase in persister cell resuscitation upon nutrient supply. Viable cell counts and resuscitation of rifampicin‐induced persister cells at 0 h and after 1–3 h of resuscitation. Persister cells (0 h) were used as the reference for fold‐change calculations. Values are presented as mean ± SEM. Fold‐change was calculated for each replicate relative to 0 h and then averaged. Table S3: RMF, Hpf and RaiA promote persister formation. Viable cell counts of exponential‐phase cells at OD600 = 0.8 prior to antibiotic treatment and persister levels formed after antibiotic exposure in E. coli BW25113 and its derivatives. Values are presented as mean ± SEM. For gene deletion mutants (Δrmf, Δhpf, ΔraiA and ΔhflX), fold‐change was calculated for each replicate based on persister rates (%) relative to the BW25113 WT control and then averaged. For plasmid‐based overexpression strains, fold‐change [file MBT2-19-e70352-s001.docx]

**Ribosome state distributions define *E. coli* persister physiology: ribosome state distributions link persister formation, stress condition, and resuscitation dynamics**

Hyein Kim^1^, and Sooyeon Song^1,2*^

^1^Department of Animal Science, Jeonbuk National University, 587 Baekje-Daero, Deokjin-Gu,

Jeonju-Si, Jellabuk-Do, 54896, South Korea

^2^Agriculture Convergence Technology, Jeonbuk National University, 587 Baekje-Daero, Deokjin-Gu,

Jeonju-Si, Jellabuk-Do, 54896, South Korea

*For correspondence. E-mail: [songsy@jbnu.ac.kr](mailto:songsy@jbnu.ac.kr)

Tel. (+)82 63-270-2606; Fax (82) 63-270-2612

**Running title:** Ribosome States in *E. coli* Persister cells

**Key words:** Antibiotic persistence; Ribosome sedimentation profiling; Ribosome hibernation; 100S ribosome; Bacterial resuscitation

**Table S1. Ribosome state distribution quantified by ribosome sedimentation profiling in *E. coli.*** Relative distributions of ribosomal species were determined by calculating the area under the curve (AUC) for each peak and normalizing to the total AUC of the profile. “Total” represents the integrated area of the entire ribosome sedimentation profile, and 30S, 50S, 70S, 90–100S, and polysome correspond to the AUC of each respective peak. WT persister cells were generated by growing cultures in LB to OD_600_ = 0.8, followed by rifampicin treatment (30 min), ampicillin treatment (3 h), and washing with 0.85% NaCl. Gene deletion mutants (Δ*rmf*, Δ*hpf*, Δ*raiA*, and Δ*hflX*; grown in LB supplemented with kanamycin) and plasmid-bearing strains (pCA24N-empty or gene-expressing constructs; grown in LB supplemented with chloramphenicol) were processed identically to obtain persister cells. WT persister cells are designated as 0 h and serve as the reference point for subsequent resuscitation time points (1, 2, and 3 h). Values are expressed as mean ± SEM and rounded to three decimal places.

|  | **30S**  **/Total** | **50S**  **/Total** | **70S**  **/Total** | **(90-100S)**  **/Total** | **Polysome**  **/Total** | **70S**  **/(90-100S)** |
| --- | --- | --- | --- | --- | --- | --- |
| **WT Exponential cells (OD_600_ 0.8)** | 0.017 ± 0.009 | 0.047 ± 0.003 | 0.173 ± 0.046 | 0.111 ± 0.023 | 0.041 ± 0.001 | 1.517 ± 0.184 |
| **WT Persister cells (0h)** | 0.016 ± 0.004 | 0.024 ± 0.001 | 0.088 ± 0.025 | 0.146 ± 0.015 | 0.028 ± 0.001 | 0.729 ± 0.032 |
| **WT Resuscitated persister cells-1h** | 0.038 ± 0.006 | 0.045 ± 0.002 | 0.070 ± 0.018 | 0.057 ± 0.013 | 0.019 ± 0.003 | 1.204 ± 0.065 |
| **WT Resuscitated persister cells-2h** | 0.020 ± 0.003 | 0.043 ± 0.005 | 0.132 ± 0.005 | 0.106 ± 0.002 | 0.034 ± 0.004 | 1.245 ± 0.038 |
| **WT Resuscitated persister cells-3h** | 0.017 ± 0.001 | 0.033 ± 0.002 | 0.126 ± 0.002 | 0.104 ± 0.001 | 0.035 ± 0.001 | 1.202 ± 0.006 |
| **Δ*rmf* Persister cells** | 0.031 ± 0.003 | 0.047 ± 0.003 | 0.105 ± 0.014 | 0.077 ± 0.009 | 0.033 ± 0.002 | 1.389 ± 0.127 |
| **Δ*hpf* Persister cells** | 0.028 ± 0.001 | 0.030 ± 0.001 | 0.071 ± 0.005 | 0.088 ± 0.008 | 0.034 ± 0.004 | 0.827 ± 0.046 |
| **Δ*raiA* Persister cells** | 0.024 ± 0.002 | 0.029 ± 0.002 | 0.079 ± 0.010 | 0.104 ± 0.009 | 0.032 ± 0.002 | 0.758 ± 0.060 |
| **Δ*hflX* Persister cells** | 0.019 ± 0.001 | 0.019 ± 0.001 | 0.074 ± 0.004 | 0.128 ± 0.008 | 0.029 ± 0.002 | 0.579 ± 0.009 |
| **pCA24N-empty Exponential cells** | 0.028 ± 0.007 | 0.048 ± 0.010 | 0.128 ± 0.002 | 0.099 ± 0.008 | 0.037 ± 0.004 | 1.307 ± 0.082 |
| **pCA24N-empty Persister cells** | 0.021 ± 0.004 | 0.028 ± 0.001 | 0.113 ± 0.002 | 0.130 ± 0.008 | 0.027 ± 0.003 | 0.873 ± 0.069 |
| **pCA24N-*rmf* Persister cells** | 0.006 ± 0.001 | 0.023 ± 0.005 | 0.109 ± 0.008 | 0.192 ± 0.008 | 0.022 ± 0.002 | 0.572 ± 0.059 |
| **pCA24N-*hpf* Persister cells** | 0.008 ± 0.002 | 0.023 ± 0.002 | 0.137 ± 0.003 | 0.159 ± 0.005 | 0.022 ± 0.001 | 0.862 ± 0.049 |
| **pCA24N-*raiA* Persister cells** | 0.010 ± 0.003 | 0.022 ± 0.005 | 0.126 ± 0.004 | 0.158 ± 0.006 | 0.025 ± 0.001 | 0.795 ± 0.057 |
| **pCA24N-*hflX* Persister cells** | 0.013 ± 0.002 | 0.016 ± 0.006 | 0.096 ± 0.006 | 0.178 ± 0.028 | 0.028 ± 0.003 | 0.579 ± 0.135 |

**Table S2.** **Time-dependent increase in persister cell resuscitation upon nutrient supply.** Viable cell counts and resuscitation of rifampicin-induced persister cells at 0 h and after 1–3 h of resuscitation. Persister cells (0 h) were used as the reference for fold-change calculations. Values are presented as mean ± SEM. Fold-change was calculated for each replicate relative to 0 h and then averaged.

|  | **Viable cells**  **(CFU/mL)** | **Fold-change** | **70S-to-100S AUC ratios** |
| --- | --- | --- | --- |
| **Exponential cells** | (6.8 ± 0.2) × 10^8^ | - | 1.517 ± 0.184 |
| **Persister cells (0 h)** | (5.9 ± 0.5) × 10^8^ | 1.0 | 0.729 ± 0.032 |
| **1 h** | (5.1 ± 0.5) × 10^9^ | 7.9 ± 0.7 | 1.204 ± 0.065 |
| **2 h** | (8.0 ± 0.8) × 10^9^ | 14.6 ± 2.8 | 1.245 ± 0.038 |
| **3 h** | (1.4 ± 0.1) × 10^10^ | 29.1 ± 7.0 | 1.202 ± 0.006 |

**Table S3. RMF, Hpf, and RaiA promote persister formation.** Viable cell counts of exponential-phase cells at OD_600_ = 0.8 prior to antibiotic treatment, and persister levels formed after antibiotic exposure in *E. coli* BW25113 and its derivatives. Values are presented as mean ± SEM. For gene deletion mutants (Δ*rmf*, Δ*hpf*, Δ*raiA*, and Δ*hflX*), fold-change was calculated for each replicate based on persister rates (%) relative to the BW25113 WT control and then averaged. For plasmid-based overexpression strains, fold-change was calculated for each replicate based on persister rates (%) relative to the BW25113 pCA24N-empty control and then averaged. 70S-to-100S AUC ratios were calculated from ribosome sedimentation profiles obtained using sucrose density gradients without glutaraldehyde (−G).

|  | **Initial cells**  **(CFU/mL, pre-treatment)** | **Persister cells**  **(CFU/mL)** | **Persister**  **formation**  **(%)** | **Fold-change** | **70S-to-100S AUC ratios** |
| --- | --- | --- | --- | --- | --- |
| **BW25113 WT** | (7.6 ± 1.3) × 10^8^ | (6.6 ± 0.7) × 10^8^ | 88.7 ± 6.0 | 1.00 | 0.729 ± 0.032 |
| **Δ*rmf*** | (7.7 ± 0.4) × 10^8^ | (2.9 ± 0.8) × 10^7^ | 3.8 ± 0.9 | -25.4 ± 4.1 | 1.389 ± 0.127 |
| **Δ*hpf*** | (2.4 ± 0.2) × 10^8^ | (1.1 ± 0.2) × 10^7^ | 4.5 ± 0.8 | -20.8 ± 2.9 | 0.827 ± 0.046 |
| **Δ*raiA*** | (4.6 ± 1.9) × 10^8^ | (7.6 ± 0.3) × 10^7^ | 16.3 ± 0.5 | -5.5 ± 0.5 | 0.758 ± 0.060 |
| **Δ*hflX*** | (7.2 ± 0.6) × 10^8^ | (6.3 ± 0.8) × 10^8^ | 86.7 ± 4.6 | -1.0 ± 0.1 | 0.579 ± 0.009 |
| **pCA24N**  **-empty** | (5.5 ± 0.1) × 10^8^ | (2.6 ± 0.0) × 10^8^ | 48.0 ± 1.2 | 1.00 | 0.873 ± 0.069 |
| **pCA24N**  **-*rmf*** | (4.8 ± 0.3) × 10^8^ | (3.5 ± 0.3) × 10^8^ | 71.6 ± 2.3 | 1.5 ± 0.1 | 0.572 ± 0.059 |
| **pCA24N**  **-*hpf*** | (5.2 ± 0.4) × 10^8^ | (3.2 ± 0.3) × 10^8^ | 62.2 ± 0.3 | 1.3 ± 0.1 | 0.862 ± 0.049 |
| **pCA24N**  **-*raiA*** | (4.2 ± 0.3) × 10^8^ | (2.6 ± 0.4) × 10^8^ | 61.6 ± 2.7 | 1.3 ± 0.1 | 0.795 ± 0.057 |
| **pCA24N**  **-*hflX*** | (7.6 ± 0.8) × 10^8^ | (3.8 ± 1.0) × 10^8^ | 48.6 ± 8.0 | 1.0 ± 0.2 | 0.579 ± 0.135 |

**Table S4. Distinct levels of persister formation under different inducing conditions.** Viable cell counts and persister levels at 0 h in exponential cells and persister populations generated under different inducing conditions. Values are presented as mean ± SEM. Persister rates (%) were calculated as the percentage of persister CFU relative to total viable CFU. Rif, rifampicin; Tetra, tetracycline. 70S-to-100S AUC ratios were calculated from ribosome sedimentation profiles obtained using glutaraldehyde-fixed (+G) sucrose density gradients.

|  | **Initial cells**  **(CFU/mL, pre-treatment)** | **Persister cells**  **(CFU/mL)** | **Persister**  **formation**  **(%)** | **70S-to-100S AUC ratios** |
| --- | --- | --- | --- | --- |
| **Exponential cells** | (6.8 ± 0.2) × 10^8^ | - | - | 3.212 ± 0.303 |
| **Natural persister cells** | (4.3 ± 0.1) × 10^8^ | (1.3 ± 0.1) × 10^3^ | 0.003 ± 0.0001 | 0.098 ± 0.004 |
| **Starvation-induced persister cells** | (1.4 ± 0.1) × 10^9^ | (8.0 ± 2.7) × 10^6^ | 0.6 ± 0.2 | 0.000 ± 0.000 |
| **Rif-induced persister cells** | (4.7 ± 0.2) × 10^8^ | (2.3 ± 0.7) × 10^7^ | 5.0 ± 1.2 | 0.186 ± 0.095 |
| **CCCP-induced persister cells** | (4.1 ± 0.2) × 10^8^ | (7.5 ± 0.1) × 10^7^ | 18.6 ± 1.1 | 0.769 ± 0.191 |
| **Tetra-induced persister cells** | (4.7 ± 0.4) × 10^8^ | (4.5 ± 0.2) × 108 | 96.8 ± 11.9 | 0.787 ± 0.044 |

**Table S5. Ribosome sedimentation profiling–based analysis of ribosome states in persister cells induced by different stresses.** Relative distributions of ribosomal species were determined by calculating the area under the curve (AUC) for each peak and normalizing to the total AUC of the profile. “Total” represents the integrated area of the entire ribosome sedimentation profile, and 30S, 50S, 70S, 90–100S, and polysome correspond to the AUC of each respective peak. Persister cells were induced using rifampicin–ampicillin (Rif-induced persister cells), carbonyl cyanide m-chlorophenyl hydrazone–ampicillin (CCCP-induced persister cells), tetracycline–ampicillin (Tetra-induced persister cells), or prolonged starvation for 6days (Starvation-induced persister cells), as indicated. Ribosome sedimentation profiling was performed using sucrose density gradients prepared either without glutaraldehyde (−G) or with glutaraldehyde fixation (+G). Ribosome sedimentation profiling data were quantified using the area under the curve (AUC) method, and values are expressed as ratios relative to the total ribosome signal. Values are expressed as mean ± SEM. All values are rounded to three decimal places.

|  |  | **Exponential cells** | **Natural persister cells** | **Starvation-induced persister cells** | **Rif-induced persister cells** | **CCCP-induced persister cells** | **Tetra-induced persister cells** |
| --- | --- | --- | --- | --- | --- | --- | --- |
| **+G** | **(30-50S)/ Total** | 0.114 ± 0.001 | 0.026 ± 0.005 | 0.000 ± 0.000 | 0.056 ± 0.023 | 0.050 ± 0.004 | 0.040 ± 0.001 |
|  | **70S/ Total** | 0.159 ± 0.003 | 0.022 ± 0.001 | 0.000 ± 0.000 | 0.041 ± 0.015 | 0.058 ± 0.006 | 0.063 ± 0.001 |
|  | **(90-100S)/ Total** | 0.050 ± 0.006 | 0.225 ± 0.002 | 0.228 ± 0.075 | 0.245 ± 0.045 | 0.081 ± 0.011 | 0.081 ± 0.006 |
|  | **Polysome/ Total** | 0.041 ± 0.001 | 0.238 ± 0.006 | 0.235 ± 0.095 | 0.128 ± 0.006 | 0.210 ± 0.003 | 0.214 ± 0.006 |
|  | **70S/ (90-100S)** | 3.212 ± 0.303 | 0.098 ± 0.004 | 0.000 ± 0.000 | 0.186 ± 0.095 | 0.769 ± 0.191 | 0.787 ± 0.044 |
| **-G** | **30S/ Total** | 0.017 ± 0.009 | 0.030 ± 0.001 | 0.045 ± 0.019 | 0.018 ± 0.004 | 0.097 ± 0.014 | 0.005 ± 0.003 |
|  | **50S/ Total** | 0.047 ± 0.003 | 0.030 ± 0.001 | 0.031 ± 0.015 | 0.028 ± 0.004 | 0.256 ± 0.009 | 0.018 ± 0.009 |
|  | **70S/ Total** | 0.173 ± 0.046 | 0.036 ± 0.001 | 0.018 ± 0.006 | 0.088 ± 0.018 | 0.020 ± 0.019 | 0.124 ± 0.005 |
|  | **(90-100S)/ Total** | 0.111 ± 0.023 | 0.038 ± 0.001 | 0.020 ± 0.004 | 0.138 ± 0.014 | 0.002 ± 0.002 | 0.172 ± 0.009 |
|  | **Polysome/ Total** | 0.041 ± 0.001 | 0.043 ± 0.002 | 0.039 ± 0.010 | 0.028 ± 0.001 | 0.000 ± 0.000 | 0.028 ± 0.002 |
|  | **70S/ (90-100S)** | 1.517 ± 0.184 | 0.937 ± 0.027 | 0.814 ± 0.141 | 0.729 ± 0.032 | 8.595 ± 8.385 | 0.728 ± 0.066 |

**Table S6. Quantitative analysis of ribosome state distribution and time-dependent resuscitation of natural persister cells in *E. coli* EHEC O157:H7.** Relative distributions of ribosomal species were determined by calculating the area under the curve (AUC) for each peak obtained from ribosome sedimentation profiling and normalizing to the total AUC of the profile. “Total” represents the integrated area of the entire ribosome sedimentation profile, and 30S, 50S, 70S, 90–100S, and polysome correspond to the AUC of each respective peak. WT persister cells were generated by growing cultures in LB to OD_600_ = 0.8, followed by ampicillin treatment (3 h) and washing with 0.85% NaCl. WT persister cells are designated as 0 h and serve as the reference point for subsequent resuscitation time points (1 and 3 h). Viable cell counts and resuscitation of natural persister cells at 0 h and after 1 and 3 h of resuscitation were analyzed. Persister cells (0 h) were used as the reference for fold-change calculations. Fold-change was calculated for each replicate relative to 0 h and then averaged. Values are presented as mean ± SEM and rounded to three decimal places.

**A. Ribosome state distribution quantified by ribosome sedimentation profiling**

|  | **30S**  **/Total** | **50S**  **/Total** | **70S**  **/Total** | **(90-100S)**  **/Total** | **Polysome**  **/Total** | **70S**  **/(90-100S)** |
| --- | --- | --- | --- | --- | --- | --- |
| **EHEC Exponential cells (OD_600_ 0.8)** | 0.029 ± 0.004 | 0.069 ± 0.008 | 0.215 ± 0.006 | 0.163 ± 0.009 | 0.029 ± 0.001 | 1.325 ± 0.058 |
| **EHEC Persister cells (0h)** | 0.000 ± 0.000 | 0.000 ± 0.000 | 0.233 ± 0.031 | 0.344 ± 0.045 | 0.000 ± 0.000 | 0.689 ± 0.089 |
| **EHEC Resuscitated persister cells-1h** | 0.050 ± 0.001 | 0.061 ± 0.004 | 0.062 ± 0.003 | 0.052 ± 0.000 | 0.050 ± 0.001 | 1.186 ± 0.056 |
| **EHEC Resuscitated persister cells-3h** | 0.021 ± 0.009 | 0.067 ± 0.013 | 0.224 ± 0.004 | 0.174 ± 0.010 | 0.017 ± 0.002 | 1.297 ± 0.055 |

**B. Time-dependent resuscitation of natural persister cells.**

|  | **Viable cells**  **(CFU/mL)** | **Fold-change** | **70S-to-100S AUC ratios** |
| --- | --- | --- | --- |
| **EHEC Exponential cells (OD_600_ 0.8)** | (7.9 ± 0.5) × 10^8^ | - | 0.689 ± 0.089 |
| **EHEC Persister cells (0 h)** | (1.1 ± 0.1) × 10^7^ | 1.0 | 1.186 ± 0.056 |
| **1 h** | (1.3 ± 0.1) × 10^7^ | 1.2 ± 0.1 | 1.297 ± 0.055 |
| **3 h** | (7.0 ± 1.2) × 10^8^ | 64.1 ± 10.7 | 1.380 ± 0.085 |

**Table S7. Statistical validation of the AUC_70S/AUC_100S ratio as a quantitative index across diverse *E. coli* populations.** Individual AUC_70S/AUC_100S ratios from all experimental conditions examined in this study (N = 87 total) are listed alongside their corresponding state assignments (non-persister vs. persister). State assignments were determined by experimental context: exponential-phase cells and resuscitated cells were designated as non-persister-state (n = 30); all antibiotic- or stress-induced populations were designated as persister-state (n = 57). Ratios were obtained from ribosome sedimentation profiles using both laboratory (BW25113) and clinical (*E. coli* O157:H7) strains. Statistical analysis using an unpaired t-test (two-tailed) confirmed a highly significant difference between the two groups (persister-state: 0.709 ± 0.375 vs. non-persister-state: 1.488 ± 0.619, P < 0.0001). Furthermore, a point-biserial correlation confirmed a strong and significant negative association between the AUC_70S/AUC_100S ratio and the persister state (r = –0.621, P < 0.0001). The single biological outlier, Δ*rmf* persister cells (ratio = 1.389), is consistent with the inability of this mutant to form canonical 100S-enriched ribosome states under the tested conditions. All underlying data are consistent with the distributions shown in **Figure S1**.

| Sample | Strain | Condition | State | Gradient | Repeat | AUC_70S  /AUC_100S |
| --- | --- | --- | --- | --- | --- | --- |
| BW WT Exponential (-G) | BW25113 | Exponential | Non-persister | -G | #1 | 1.41 |
|  |  |  |  |  | #2 | 1.87 |
|  |  |  |  |  | #3 | 1.27 |
| BW pCA24N-empty Exponential (-G) | BW25113 | Exponential | Non-persister | -G | #1 | 1.353 |
|  |  |  |  |  | #2 | 1.42 |
|  |  |  |  |  | #3 | 1.148 |
| BW WT Resuscitation 1h | BW25113 | Resuscitation | Non-persister | -G | #1 | 1.281 |
|  |  |  |  |  | #2 | 1.074 |
|  |  |  |  |  | #3 | 1.26 |
| BW WT Resuscitation 2h | BW25113 | Resuscitation | Non-persister | -G | #1 | 1.17 |
|  |  |  |  |  | #2 | 1.296 |
|  |  |  |  |  | #3 | 1.268 |
| BW WT Resuscitation 3h | BW25113 | Resuscitation | Non-persister | -G | #1 | 1.211 |
|  |  |  |  |  | #2 | 1.19 |
|  |  |  |  |  | #3 | 1.205 |

**Table S7 (Continued)**

| Sample | Strain | Condition | State | Gradient | Repeat | AUC_70S  /AUC_100S |
| --- | --- | --- | --- | --- | --- | --- |
| BW WT Exponential (+G) | BW25113 | Exponential | Non-persister | +G | #1 | 2.664 |
|  |  |  |  |  | #2 | 3.262 |
|  |  |  |  |  | #3 | 3.711 |
| BW WT Persister (-G) | BW25113 | Rif-induced | Persister | -G | #1 | 0.734 |
|  |  |  |  |  | #2 | 0.671 |
|  |  |  |  |  | #3 | 0.782 |
| BWΔ*rmf* Persister | BW25113 | Rif-induced | Persister | -G | #1 | 1.411 |
|  |  |  |  |  | #2 | 1.916 |
|  |  |  |  |  | #3 | 1.112 |
|  |  |  |  |  | #4 | 1.125 |
|  |  |  |  |  | #5 | 1.206 |
|  |  |  |  |  | #6 | 1.561 |
| BW Δ*hpf* Persister | BW25113 | Rif-induced | Persister | -G | #1 | 0.853 |
|  |  |  |  |  | #2 | 0.889 |
|  |  |  |  |  | #3 | 0.913 |
|  |  |  |  |  | #4 | 0.601 |
|  |  |  |  |  | #5 | 0.864 |
|  |  |  |  |  | #6 | 0.843 |
| BW Δ*raiA* Persister | BW25113 | Rif-induced | Persister | -G | #1 | 0.886 |
|  |  |  |  |  | #2 | 0.851 |
|  |  |  |  |  | #3 | 0.921 |
|  |  |  |  |  | #4 | 0.697 |
|  |  |  |  |  | #5 | 0.586 |
|  |  |  |  |  | #6 | 0.611 |

**Table S7 (Continued)**

| Sample | Strain | Condition | State | Gradient | Repeat | AUC_70S  /AUC_100S |
| --- | --- | --- | --- | --- | --- | --- |
| BW Δ*hflX* Persister | BW25113 | Rif-induced | Persister | -G | #1 | 0.561 |
|  |  |  |  |  | #2 | 0.593 |
|  |  |  |  |  | #3 | 0.582 |
| BW pCAN-empty Persister | BW25113 | Rif-induced | Persister | -G | #1 | 0.959 |
|  |  |  |  |  | #2 | 0.923 |
|  |  |  |  |  | #3 | 0.737 |
| BW pCAN-*rmf* Persister | BW25113 | Rif-induced | Persister | -G | #1 | 0.654 |
|  |  |  |  |  | #2 | 0.605 |
|  |  |  |  |  | #3 | 0.457 |
| BW pCAN-*hpf* Persister | BW25113 | Rif-induced | Persister | -G | #1 | 0.928 |
|  |  |  |  |  | #2 | 0.892 |
|  |  |  |  |  | #3 | 0.766 |
| BW pCAN-*raiA* Persister | BW25113 | Rif-induced | Persister | -G | #1 | 0.825 |
|  |  |  |  |  | #2 | 0.855 |
|  |  |  |  |  | #3 | 0.705 |
| BW pCAN-*hflX* Persister | BW25113 | Rif-induced | Persister | -G | #1 | 0.846 |
|  |  |  |  |  | #2 | 0.408 |
|  |  |  |  |  | #3 | 0.484 |
| BW Natural Persister (+G) | BW25113 | Natural persister | Persister | +G | #1 | 0.091 |
|  |  |  |  |  | #2 | 0.099 |
|  |  |  |  |  | #3 | 0.104 |
| BW Starvation Persister (+G) | BW25113 | Starvation-induced | Persister | +G | #1 | 0 |
|  |  |  |  |  | #2 | 0 |
|  |  |  |  |  | #3 | 0 |

**Table S7 (Continued)**

| Sample | Strain | Condition | State | Gradient | Repeat | AUC_70S  /AUC_100S |
| --- | --- | --- | --- | --- | --- | --- |
| BW Rif-induced Persister (+G) | BW25113 | Rif-induced | Persister | +G | #1 | 0.050 |
|  |  |  |  |  | #2 | 0.139 |
|  |  |  |  |  | #3 | 0.369 |
| BW CCCP-induced Persister (+G) | BW25113 | CCCP-induced | Persister | +G | #1 | 1.150 |
|  |  |  |  |  | #2 | 0.633 |
|  |  |  |  |  | #3 | 0.528 |
| BW Tetra-induced Persister (+G) | BW25113 | Tetra-induced | Persister | +G | #1 | 0.842 |
|  |  |  |  |  | #2 | 0.820 |
|  |  |  |  |  | #3 | 0.699 |
| EHEC Natural Persister | O157:H7 | Natural persister | Persister | -G | #1 | 0.845 |
|  |  |  |  |  | #2 | 0.537 |
|  |  |  |  |  | #3 | 0.684 |
| EHEC Exponential | O157:H7 | Exponential | Non-persister | -G | #1 | 1.218 |
|  |  |  |  |  | #2 | 1.342 |
|  |  |  |  |  | #3 | 1.416 |
| EHEC Resuscitation 1h | O157:H7 | Resuscitation | Non-persister | -G | #1 | 1.077 |
|  |  |  |  |  | #2 | 1.220 |
|  |  |  |  |  | #3 | 1.262 |
| EHEC Resuscitation 3h | O157:H7 | Resuscitation | Non-persister | -G | #1 | 1.292 |
|  |  |  |  |  | #2 | 1.204 |
|  |  |  |  |  | #3 | 1.394 |

**Supplementary figure**

**Figure S1. Statistical analysis of the AUC_70S/AUC_100S ratio across diverse *E. coli* populations.** The AUC_70S/AUC_100S ratio was analyzed to evaluate its consistency as a quantitative marker for the 100S-enriched ribosome state.

**A**. Distribution of AUC_70S/AUC_100S ratios in non-persister (n =27) and persister-state (n = 57) samples across all experimental conditions, including laboratory (BW25113) and clinical (*E. coli* O157:H7) strains. Box plots represent the median and interquartile range (IQR), with whiskers extending to 1.5 x IQR. Individual data points are shown as black circles (N =84 total). Persister-state samples exhibited significantly lower ratios compared to non-persister samples (P < 0.0001, unpaired t-test (two-tailed).

**B.**  Point-biserial correlation analysis between the AUC_70S/AUC_100S ratio and the bacterial state (non-persister vs. persister). A statistically significant negative association was confirmed (r = -0.603, P < 0.0001). *rmf* mutant persister samples (red circle; #1 ratio = 1.411, #2 ratio = 1.916, #3 ratio = 1.112, #4 ratio = 1.125, #5 ratio = 1.206, and #6 ratio = 1.561) are indicated as a biological outlier, consistent with its inability to form canonical 100S ribosomes under the conditions tested. All underlying data are provided in **Supplementary Table S7**.

**Figure S2. Effect of glutaraldehyde stabilization on ribosome sedimentation profiles of diverse *E. coli* persister populations.**

**A. Ribosome sedimentation profiles diverse persister populations without glutaraldehyde stabilization.** Ribosome sedimentation profiles of BW25113 WT exponential-phase cells and the diverse persister populations described in Fig. 4A. The same samples used in Fig. 4 were analyzed on 5–20% sucrose density gradients without glutaraldehyde stabilization and monitored at OD_260_.

**B. AUC analysis of ribosome distributions without glutaraldehyde stabilization.** Quantification of ribosomal species by area under the curve (AUC) analysis of the profiles shown in A., comparing ribosome distributions among the indicated persister populations under non-stabilized conditions
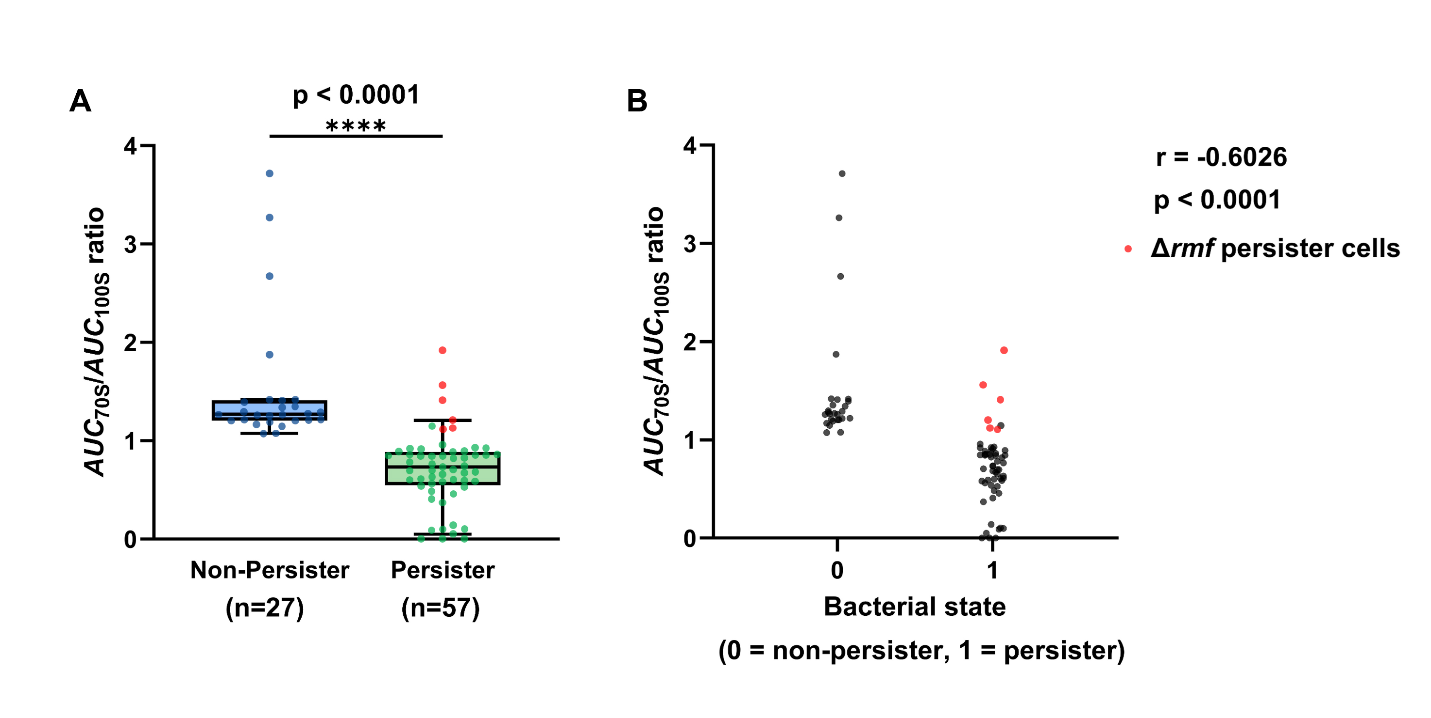


**Figure S1**

**
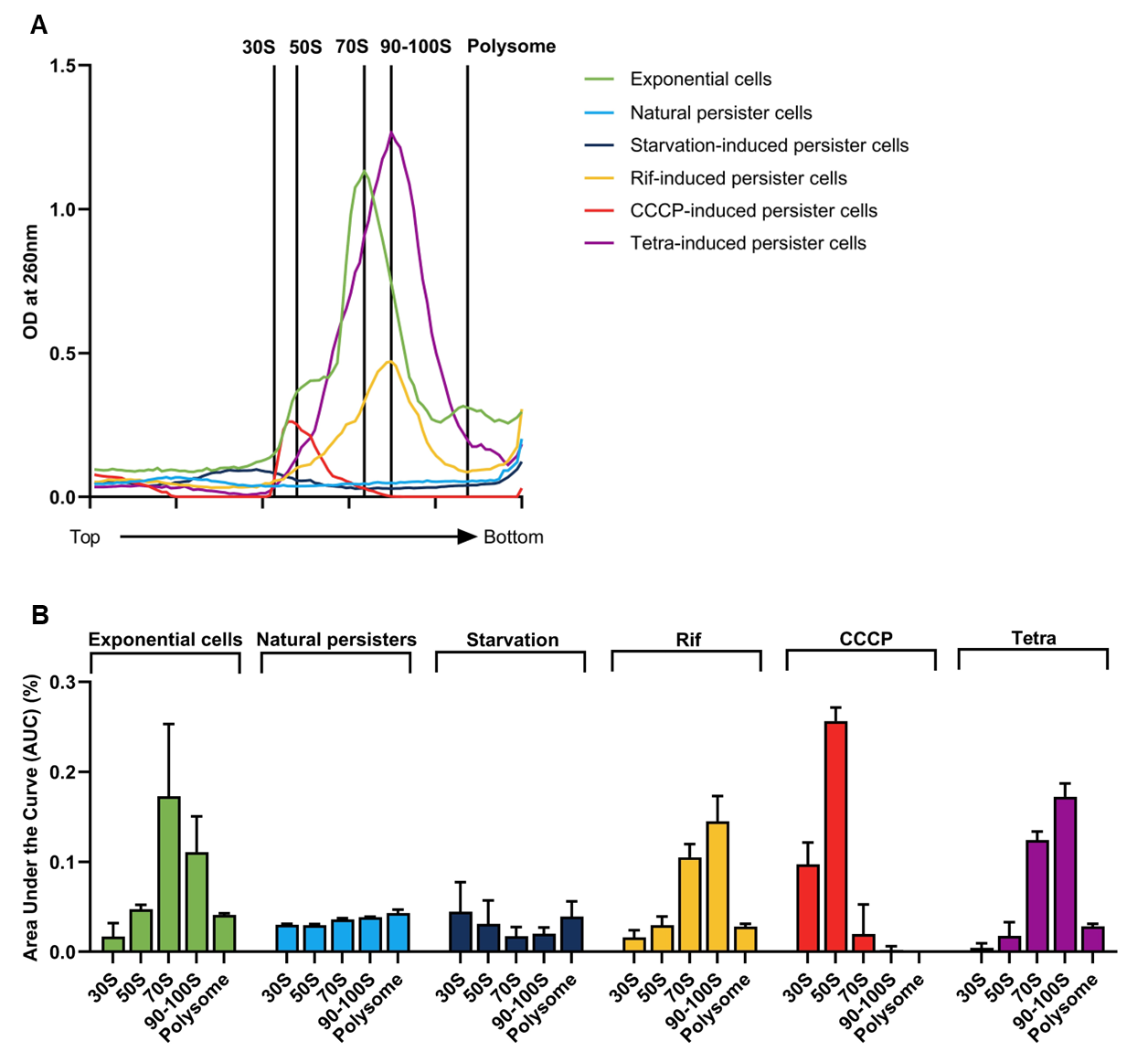
**

**Figure S2**
